# Implementation strategies to increase tobacco treatment in mental health settings: a systematic review

**DOI:** 10.1186/s12888-025-07248-7

**Published:** 2025-10-08

**Authors:** Corinne N. Kacmarek, Anthony A. Vivino, Hannah C. Smith, Julie Kreyenbuhl

**Affiliations:** 1 VISN 5 Capitol Health Care Network, Mental Illness Research, Education, and Clinical Center (MIRECC), Veterans Health Administration, Baltimore, MD USA; 2 Department of Psychiatry, Division of Psychiatric Services Research, University of Maryland School of Medicine, Baltimore, MD USA; 3 Department of Psychological and Brain Sciences, University of Delaware, Newark, DE, USA; 4 Department of Psychiatry, Division of Addiction Research and Treatment, University of Maryland School of Medicine, Baltimore, MD USA

**Keywords:** Tobacco treatment, Mental health, Health care provider, Implementation science, Behavior change, Systematic review, Outpatient mental health, Inpatient mental health

## Abstract

**Background:**

Tobacco smoking rates have declined for the general population, but remain high for individuals with mental illness. Increasing access to tobacco treatment interventions in mental health settings is essential to address this health inequity. This systematic review aimed to determine how implementation strategies change mental health provider delivery of tobacco interventions.

**Methods:**

We searched PubMed, PsycInfo, CINAHL, Ovid MedLine, Embase, and grey literature from inception through December 31, 2024. Implementation strategies, behavior change mechanisms, and outcomes were categorized using the Expert Recommendations for Implementing Change (ERIC) taxonomy, capability-opportunity-motivation behavioral model, and Reach, Effectiveness, Adoption, Implementation, Maintenance framework, respectively. Changes in provider adoption of implementation activities or the 5As (*Ask*,* Advise*,* Assess*,* Assist*,* Arrange)*, and changes in reach of 5As to patients, were primary outcomes and reported as a percentage change after, compared to before, the implementation strategy.

**Results:**

Twenty of 786 studies met inclusion criteria. Half (55%) were at serious risk of bias due to confounding. For behavior change mechanisms, all outpatient studies addressed clinician capability, and all inpatient studies addressed clinician opportunity to provide tobacco interventions. Reach was the most common outcome evaluated, with the largest absolute increases in proportion of patients being *Assisted* with referrals across both outpatient (42%) and inpatient (11%) settings after implementation. ERIC domains that maximized nicotine replacement therapy (NRT) delivery differed by setting: Strategies used in the *engaging consumers* domain were associated with 16% NRT increases in outpatient settings and *supporting clinicians* and *changing infrastructure* increased NRT by 20% in inpatient settings.

**Conclusion:**

Interactions between setting, implementation domain, and outcomes improved reach of tobacco interventions to varying degrees in mental health settings, but formal analyses are needed to confirm hypothesized moderators (inpatient vs. outpatient) and mediators (capability, opportunity, motivation) of change. Strategies that *engage consumers* in outpatient settings and *change infrastructure* in inpatient settings may be particularly useful for improving reach of tobacco medications. Future implementation trials must monitor changes in the gold-standard tobacco interventions (medication + counseling) to maximize the clinical impact of provider practice improvements. Clinical-research partnerships are also needed to explore feasible ways to evaluate smoking changes in real-world clinical settings.

**Supplementary Information:**

The online version contains supplementary material available at 10.1186/s12888-025-07248-7.

## Background

After 50 years of tobacco-control efforts, tobacco smoking remains a leading cause of preventable death worldwide and the leading cause of disability in North America and the United Kingdom (UK) [1]. Globally, smoking prevalence has decreased from 43.1% in 1990 to 13.7% in 2015 as a result of smoke-free policies, social marketing that has de-normalized smoking, and development and dissemination of evidence-based tobacco interventions [1, 2]. Unfortunately, these political, social, and medical efforts have not been sufficient to reduce smoking among those who meet criteria for a mental illness according to the American Psychiatric Association’s Diagnostic and Statistical Manual [3–5]. In the United States (US), individuals with mental illness smoke at double the rate of those without mental illness [4]. As a result, the smoking disparity has continued to widen between those with and without mental illness [6, 7]. To reduce this disparity, efforts designed to reduce demand for tobacco and expand access to tobacco interventions must prioritize populations with mental illness [8].

Nearly 70% of individuals, including those with mental illness, want to quit [2, 9, 10]. Though people with mental illness have higher nicotine dependence and lower abstinence rates than those without mental illness, they are also more likely to use tobacco treatment compared to those without mental illness [2]. In addition to reducing mortality and disability, a systematic review and meta-analysis found that individuals with mental illness who quit smoking experienced reduced depression − 0.39 (95% CI [− 0.63 to − 0.14]), reduced mixed anxiety and depression (− 0.2, 95% CI[− 1.07 to 0.65]), improved psychological quality of life (0.40, 95% CI[− 0.03 to 0.83]), and improved positive affect (0.68, 95% CI[0.24 to 1.12]) [11].

Tobacco interventions that address the physical, behavioral, social, and psychological components of nicotine addiction are essential to sustaining tobacco cessation for those with and without mental illness [2, 12]. According to the US Public Health Service’s Tobacco Use and Dependence Guidelines, interventions like the 5As – *Asking* about smoking, *Advising* quitting, *Assessing* interest in quitting, *Assisting* with medication, referrals, or behavioral counseling, and *Arranging* follow-up (5As) – can increase quit attempts for individuals ready to quit within 30 days; and counseling that includes *Repeatedly* discussing *Relevance* of quitting, *Risks* of smoking, *Rewards* of quitting, and *Roadblocks* to quitting (5Rs) can increase quit attempts for individuals contemplating change [2, 12]. In addition, there are six US Food and Drug Administration (FDA)-approved medications: nicotine replacement therapies (NRT; nasal spray, gum, lozenge, patch), bupropion, and varenicline (formerly Chantix). These medications double or even quadruple quit rates compared to placebo [12] and are safe and effective for individuals with mental illness [13–15].

Given the high rates of smoking among those with mental illness, mental health providers work with a disproportionally large number of smokers. People with mental illness have more frequent contact with their mental health providers compared to other health care providers, making mental health providers uniquely suited to deliver tobacco interventions like the 5As, 5Rs, and tobacco medication [16]. However, many are often reluctant to do so [17–19]. Individuals diagnosed with certain mental illnesses, particularly schizophrenia and other psychotic disorders, are less likely to be advised to quit and receive quit medication compared to individuals without those conditions [11, 20–22], and psychiatrists are less likely to prescribe tobacco medication than primary care providers [23].

Across three systematic reviews [17–19] covering 77 unique studies, common barriers to delivering tobacco interventions in mental health settings emerged. Most mental health providers report inadequate training in tobacco interventions, competing clinical demands, and the belief that patients do not want to or are unable to quit [17–19]. These skill, knowledge, attitude, and resource barriers create discomfort for mental health providers and lead them to avoid discussing smoking or, in some cases, to enable it. Thus, evidence-based tobacco interventions are underutilized in mental health settings despite a demonstrated need for their use [24]. From a public health perspective, increasing patient quit *attempts* and quit *success* are essential to reducing smoking, with the former achieved by expanding the reach of tobacco interventions and the latter by providing interventions at an intensity commensurate with level of dependence [2]. Understanding barriers to provider delivery of tobacco interventions is the first step to reducing the burden of smoking that disproportionally impacts those with mental illness.

Efforts are needed to overcome barriers to tobacco intervention delivery in mental health settings. *Implementation strategies* are purposeful efforts to improve the uptake of treatment interventions (e.g., the 5As for tobacco use) by individuals qualified to deliver such interventions (e.g., health care providers) [25]. Although barriers to delivering tobacco interventions in mental health settings are well-documented, it is unclear which implementation strategies can improve provider delivery of these interventions. Lindson and colleagues (2021) reviewed findings from 81 randomized controlled trials that evaluated the impact of implementation strategies in primary care. They found that the strategies like provider education increased provider *Asking* about smoking, *Advising* to quit, *Assisting* with quit counseling, and *Assisting* with self-help materials. They also found that combining provider education with other strategies like external support and treatment decisions-making aids resulted in increased patient quit rates (RR 1.70, 95% CI 1.27 to 2.27), but had an unclear impact on providers *Asking* about smoking and *Assisting* patient with tobacco medication [26]. In a Cochrane Review, Thomas and colleagues [27] reviewed the impact of the following implementation strategies: introducing a system to record patient smoking status; providing education, resources, and feedback to providers; budgeting resources to help providers deliver tobacco interventions; identifying a dedicated staff member to provide tobacco interventions; introducing rules to restrict smoking; integrating tobacco interventions into routine care; and reimbursing providers for delivering tobacco interventions [12]. Various combinations of these strategies improved provider *Asking* about tobacco use, *Advising* quitting, *Assisting* with counseling, and *Assisting* with quit line referral and enrollment, but had a mixed impact on provider *Assisting* with medication and with quitting. Tildy and colleagues [28] narratively reviewed findings from 49 studies that evaluated the impact of implementation strategies delivered on a state or national scale in primary care. They found that adding tobacco medication to formulary increased provider *Advice* to quit and *Assisting* with quit pharmacotherapy, changing the EHR system increased providers *Assisting* with quit pharmacotherapy and patient quit rates, and training providers increased provider *Advice* to quit, provider *Assistance* with quit pharmacotherapy, and patient quit rates when combined with preparing patients to be active treatment participants [28].

In sum, systematic reviews conclude that different strategies can improve delivery of tobacco interventions and even increase patient quit rates in primary care. The aim of this systematic review was to characterize implementation strategies designed to improve provider delivery of the 5As, specifically, in mental health settings due to disproportionally high smoking and low tobacco treatment rates in these settings. Results can inform ongoing research efforts that aim to reduce smoking-related health inequities between individuals with and without mental illness.

## Methods

### Inclusion criteria

We used systematic review methodology [29]. Review procedures and reporting follow the PRISMA checklist for systematic review protocols [30] (clinical trial number: not applicable). This review was registered in PROSPERO (#CRD42024505228) and its protocol can be accessed at https://www.crd.york.ac.uk/PROSPERO/#searchadvanced.^1^ This systematic review considered experimental, quasi-experimental, and observational studies with full text available in English. Opinion papers, systematic reviews, protocol papers, and studies that only report qualitative data were not considered for inclusion.

#### Participants

Studies conducted anywhere in the world in any type of mental health setting (inpatient, outpatient, or residential) with data about mental health provider tobacco intervention practices, mental health patient smoking behavior, or both were considered for inclusion. This review did not include studies using implementation strategies for tobacco interventions in primary care, which has been the focus of prior reviews [26–28]. Studies that exclusively treated substance use disorders were also not included because tobacco treatment patterns differ between those diagnosed with substance use disorders and those diagnosed with mental health disorders [23]. Finally, we did not include studies that evaluated tobacco interventions delivered by research staff external to a clinic because the objective of this review was to evaluate strategies that can change how providers internal to a clinic deliver tobacco interventions.

#### Implementation strategy

We use the term *intervention* to refer to evidence-based tobacco treatment interventions and *implementation strategy* to refer to the activities designed to improve delivery of tobacco interventions. Though the term *intervention* is used by the population, intervention, comparator, outcome (PICO) framework, we examined *implementation strategies* to determine inclusion as well as to align with our research objective and reporting recommendations for implementation-focused studies [31]. We named and defined implementation strategies according to the Expert Recommendations for Implementation Change (ERIC) taxonomy [32], which provides a list of mutually exclusive implementation strategies [32]^2^. We then used the Behavior Change Wheel (BCW) [31, 33] to identify potential mechanisms of change using the COM-B model of behavior, which posits that one’s physical and psychological capabilities and social and environmental opportunities impact motivation and behavior [33]. The Consolidated Framework for Implementation Research (CFIR) recommends using the BCW to identify barriers and facilitators of behavior change in its *individual characteristics* domain [34]. Additionally, the BCW has been applied in real-world settings to categorize components of tobacco control strategies [33].

#### Comparator

Studies needed to compare delivery of the 5As or 5Rs before and after an implementation strategy.

#### Outcomes

Outcomes were evaluated according to the Reach, Effectiveness, Adoption, Implementation, and Maintenance (RE-AIM) framework. The outcomes were as follows: Reach is the proportion of patients who received the 5As, Effectiveness is the public health impact of the 5As (i.e., quit rates), Adoption^3^ is the proportion of providers who take up implementation activities or the 5As, Implementation is fidelity to key components of tobacco interventions, and Maintenance is the sustainability of Reach, Adoption, or Effectiveness outcomes [35, 37].

Primary outcomes were [1] absolute changes in reach of the *Asking* all psychiatric patients whether they smoke tobacco and changes in reach of the remaining 4As (*Advise*,* Assess*,* Assist*,* and Arrange*) to patients who smoke and [2] provider adoption of the 5As [35, 36]. The 5As were selected as the primary outcome due to their evidence base for identifying and treating smoking [2, 12], ability to be integrated into routine health care practices [12], and inclusion in prior systematic reviews of tobacco intervention implementation strategies [26, 28].

### Search strategy

The search strategy identified published studies and grey literature. A search of the Cochrane Databases of Systematic Reviews, PubMed, PsycInfo, CINAHL, Ovid MedLine, Embase, and PROSPERO ruled-out the existence of published or underway systematic reviews on this topic. Then, a three-step search strategy identified eligible studies. First, syntax was iteratively piloted in PubMed to optimize search parameters and compare search results with some studies on this topic that were already known to the primary author (CK). Second, text in the titles, abstracts and the index terms of studies deemed relevant were used to refine the syntax. Third, the syntax was finalized and used to conduct the literature search. Using Boolean logic, the final syntax searched for the following combinations of key words in titles and abstracts and was adapted for each database using the Systematic Review Accelerator’s Polyglot Search Translator [38]: ((tobacco) or (smok*)) AND ((quit) OR (cessation) OR (treatment)) AND ((implement*) or (strateg*) or (intervention)) AND ((mental) or (psychiatric)) AND ((provider) or (clinician) or (staff) or (personnel) or (center)). Finally, reference lists from included studies were reviewed to identify additional eligible studies.

We searched PubMed, PsycInfo, CINAHL, Ovid MedLine, Embase, and sources of grey literature from inception through February 7, 2024 and again on December 31, 2024. We identified grey literature through WorldCat, the academic repository OpenDOAR and websites from the following agencies: Academy Health, Department of Health and Human Services, Health Resources and Services Administration, the World Health Organization, Agency for Healthcare Research and Quality, and RAND.

Titles and abstracts of search results were collated by database and uploaded into Rayyan. Duplicates were removed using Rayyan’s automatic duplicate detection based on digital object identifier, study title, and first author’s last name. Titles and abstracts were screened for inclusion blindly and independently in Rayyan by authors CK and AV. CK and AV then blindly and independently reviewed the full text of studies that passed initial screening. Discrepancies were resolved through discussion after independent screening and again after independent full-text review.

### Data extraction

CK and AV extracted the following data from all studies determined to meet inclusion criteria using an a priori tool developed by CK: country, research design, participants, setting, tobacco intervention type, theoretical framework, implementation strategies, length of implementation, and main findings from RE-AIM outcomes. Data extractors identified country, participants, setting, theoretical framework, and length of implementation using language provided by the authors of each study. Coders labeled research design according to guidance from Cochrane Group [39, 40]. If tobacco intervention type and outcome were not described using terminology consistent with the 5As and RE-AIM, they were labeled by coders using US Public Health Services Tobacco Use Guidelines [12] language for the 5As and RE-AIM definitions informed by Holtrop and colleagues [37] and the Veterans Health Affairs Quality Enhancement Research Initiative implementation manual [36].

First, implementation strategies were categorized using the ERIC taxonomy. CK emailed authors of included studies, when needed, to request additional details about implementation strategies for appropriate categorization. Second, CK, AV, and HCS blindly and independently labeled implementation strategies according to the BCW using instructions provided by Michie et al. [33]. Agreement between CK and AV was 70% and between CK and HCS was 78%, which is similar to BCW agreement levels reported elsewhere [33]. CK made the final BCW determination. Unknown data for all data categories was marked accordingly. Additional studies were consulted to obtain details about the implementation strategy, only, for Correa-Fernández [41] and both included McFall et al., (2005, 2010) studies [42]. CK classified outcomes according to RE-AIM [35, 43]. Any questions that arose during data extraction were resolved through discussion.

### Data synthesis

CK evaluated risk of bias using the Risk Of Bias In Non-randomized Studies of Interventions (ROBINS-I) [39] for non-randomized studies and Risk of Bias-2 (RoB-2) for randomized controlled studies [40]. Evidence quality was not evaluated due to a lack of guidance for implementation-focused studies.

In line with Cochrane guidance, studies rated at critical risk of bias are included in tables and figures of study characteristics but not the data synthesis [39, 40]. Results will be described according to the Synthesis Without Meta-Analysis (SWiM) guidelines [44] and focus on absolute percent changes in reach and adoption of the 5As between baseline and follow-up by setting and implementation strategy domain. Reach of the 5As addresses changes in the proportion of patients receiving the 5As and adoption addresses changes in proportion of providers delivering the 5As. Outcomes are differentiated by setting (inpatient vs. outpatient) due to differences in treatment philosophy [45]. When more than one follow-up period was reported, results are reported from the longest follow-up period to emphasize the maintenance of changes, in line with the RE-AIM maintenance outcome [35, 37]. Microsoft Excel was used to generate figures summarizing results.

## Results

**Fig. 1:**
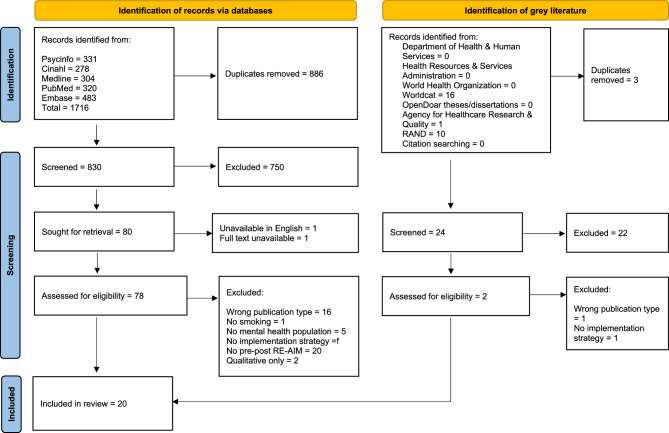
PRISMA diagram for identifying eligible studies Diagram reflects total number of articles screened on February 7, 2024 and December 31, 2024. Diagram obtained from: Page MJ, et al. BMJ 2021;372:n71. doi: 10.1136/bmj.n71. This work is licensed under CC BY 4.0. To view a copy of this license, visit https://creativecommons.org/licenses/by/4.0/

After removing duplicates, our search yielded a total of 830 studies, of which 750 were excluded during abstract screening, 78 were available for full text screening, and twenty met inclusion criteria. Of the 24 grey literature articles screened, we reviewed the full text of two and neither met the inclusion criteria. See Fig. 1for Preferred Reporting Items for Systematic Reviews and Meta-Analyses (PRISMA) diagram. During full-text screening, reviewers had 91% agreement (kappa = 0.79).

Some studies approached but did not meet our full inclusion criteria. For example, we excluded one study [46] that served a clinical population with developmental disorders and co-occurring mental illness. We also excluded a study that evaluated a national implementation strategy, State Leadership Academies, to reduce smoking among behavioral health populations throughout the US. This did not align with the aim of the present review, which was to explore implementation strategies delivered on a hospital or clinic level [47] given how setting-specific contextual and cultural factors influence an implementation strategy’s impact [34, 48, 49].

### Study characteristics

Fifteen studies were conducted in the US [50–64], three in the UK [65–67], and two in Australia [68, 69]. Five studies were randomized controlled trials (RCTs) [50, 54, 56, 57, 64]. One study was observational [55], with the remainder being experimental. Most studies used a repeated cross-sectional design [51–53, 58, 59, 61, 63, 65, 68, 69], and one used a cohort design [60]. Two studies [66, 67] used a cross-sectional design for reach outcomes and a cohort design to measure Effectiveness outcomes. A complete list of study characteristics is present in supplemental file 1.

#### Participants

Five studies collected outcomes from providers [53, 59, 60] or both patients and providers [54, 64]. Provider sample sizes ranged from 10 [60] to 1,237 [59]. Two studies included both prescribers and non-prescribers [59, 64], one only included psychiatrists [54], and one only included non-prescribers [60]. One study [55] surveyed hospital administrators on the impact of smoking bans in local mental health inpatient settings. The remainder reported patient outcomes only [50–52, 56–58, 61–63, 65–69]. Patient sample sizes ranged from 54 [58] to 30,908 [62] and were diagnostically heterogeneous, including psychotic, affective, and personality disorders. Two studies focused on a subpopulation: Veterans receiving treatment for post-traumatic stress disorder (PTSD) [56, 57].

#### Implementation strategy domains and mechanisms

Nineteen implementation programs were described across 20 studies: Two studies [53, 59] evaluated an implementation program called Taking Texas Tobacco Free (TTTF). Two others [56, 57] evaluated an implementation program that trained clinicians to integrate smoking and PTSD treatment, but the implementation strategies differed between these studies so they are each considered to have their own implementation program.

Ten studies across nine implementation programs [50, 52–54, 56, 57, 59, 60, 64, 66] were conducted in outpatient mental health settings. Ten implementation programs across ten studies [51, 55, 58, 61–63, 65, 67–69] were in a mental health inpatient setting. One [67] study was conducted in an inpatient and outpatient setting but is counted as an inpatient study because the primary outcome, reach, was only reported for inpatients. None of the eligible studies were conducted in a residential mental health setting.

The average number of individual strategies per program was 7 (*Mdn* = 6, SD = 4, range = 2–20). The most common ERIC strategy domains used in the nine outpatient programs were *train and educate stakeholders* (*n* = 9; 100%), *use evaluative and iterative strategies* (*n* = 6; 67%), and *support clinicians* (*n* = 6; 67%), followed by *develop stakeholder interrelationships* (*n* = 5; 56%), *change infrastructure* (*n* = 4; 44%), *engage consumers* (*n* = 4; 44%), *provide interactive assistance* (*n* = 2; 22%), *adapt and tailor to context* (*n* = 1; 11%), and *utilize financial strategies* (*n* = 1; 11%).

In the 10 inpatient programs, the most common strategy domains were *change infrastructure* (*n* = 10; 100%), *support clinicians* (*n* = 8; 80%), and *train and educate stakeholders* (*n* = 8; 80%), followed by *use evaluative and iterative strategies* (*n* = 5; 50%), *develop stakeholder interrelationships* (*n* = 3; 30%), *utilize financial strategies* (*n* = 3; 30%), *provide interactive assistance* (*n* = 2; 20%), and *engage consumers* (*n* = 2; 20%). *Adapt and tailor to context* was not reported in any inpatient study.

When identifying behavior change mechanisms using the BCW [33], all but one of the 19 implementation programs targeted physical and social opportunity. Of note, this program [60] focused on increasing provider delivery of training to staff. Therefore, all programs that aimed to improve tobacco intervention delivery to patients targeted both opportunity components. Most (68%, *n* = 13) programs targeted all six behavior change components. There were also differences in mechanisms by setting. All outpatient programs addressed physical and psychological capability and reflective motivation, and 89% addressed automatic motivation, physical opportunity, and social opportunity. Table 1 illustrates behavior change mechanisms by setting and implementation program. All inpatient programs addressed physical and social opportunity, 90% addressed reflective and automatic motivation, 70% addressed psychological capability, and 50% addressed physical capability.

**Table 1:** a. Outpatient implementation program COM-B mechanisms. b. Inpatient implementation program COM-B mechanisms

	Capability - physical	Capability - psychological	Motivation - Reflective	Motivation - Automatic	Opportunity - Physical	Opportunity - Social
Brunette 2015	x	x	x	x	x	x
Chen 2018	x	x	x	x	x	x
Correa-Fernández 2019 & Nitturi 2021a	x	x	x	x	x	x
Dixon 2009	x	x	x	x	x	x
Kanter Bax 2020	x	x	x	x	x	x
McFall 2005	x	x	x	x	x	x
McFall 2010	x	x	x	x	x	x
Nitturi 2021	x	x	x			
Schnoll 2023	x	x	x	x	x	x
Total	9 (100%)	9 (100%)	9 (100%)	8 (89%)	8 (89%)	8 (89%)
Carrillo 2017		x	x	x	x	x
Hollen 2010					x	x
Huddlestone 2018			x	x	x	x
Lappin 2020	x	x	x	x	x	x
Muladore 2018	x	x	x	x	x	x
Okoli 2018			x	x	x	x
Parker 2012	x	x	x	x	x	x
Scharf 2011	x	x	x	x	x	x
Scheeres 2020		x	x	x	x	x
Wye 2017	x	x	x	x	x	x
Total	5 (50%)	7 (70%)	9 (90%)	9 (90%)	10 (100%)	10 (100%)

#### Comparator

The five RCTs compared outcomes both before and after implementation efforts and to a control group [50, 54, 56, 57, 64]. All RCTs were conducted in outpatient settings. Comparators in the RCTs included the same implementation strategy delivered via a different delivery method (in-person versus virtually) [50], the same implementation strategy delivered at another site 6 months later [54], treatment as usual for tobacco use (i.e., referral to tobacco treatment) [56, 57], and provider training only [64]. The non-randomized studies used a pre-implementation period as the control group [51–53, 55, 58–63, 65–69].

#### Outcomes

Patient-level reach outcomes were reported by 13 studies and were the most commonly reported outcome [50–52, 54, 58, 61, 63–69]. Six of these studies evaluated *Asking* about tobacco use [51, 61, 65, 66, 68, 69], two evaluated changes in *Advising* quit [65, 69], none evaluated changes in *Assessing* motivation, and all 13 evaluated changes in *Assisting* in some way [50–52, 54, 58, 61, 63–69]. Of the 13 *Assisting* studies, most (*n* = 12) targeted *Assisting* with medication [50–52, 54, 58, 61, 63–65, 67–69], 2 targeted *Assisting*^4^ with counseling [51, 61], and four targeted *Assisting* with referrals to quit resources [51, 54, 65, 66]. Of the 12 *Assist* studies evaluating medications, 9 included NRT [50, 54, 58, 61, 64, 65, 67–69], three included bupropion/Wellbutrin [51, 54, 64], and five included Chantix/varenicline [50, 52, 54, 63, 64]. None of the studies monitored changes in reach of *Arranging* follow-up.

Two studies using the TTTF implementation program reported changes in provider adoption of *Asking¸ Advising*,* Assessing*, general *Assisting* (tobacco-focused medication, counseling, or referrals), and *Arranging* [53, 59]; one of these studies also evaluated changes in provider past-year attendance at tobacco education programs [53], and the other evaluated changes in the number of tobacco education sessions offered to clinicians [60]. One study reported changes in hospital-level adoption of *Assisting* with NRT and non-NRT, as well as hospital-level adoption of tobacco treatment education for clinicians [55], and seven studies [52, 54, 56, 57, 64, 66, 67] reported effectiveness outcomes.

Three studies reported other outcomes that most closely fit into the implementation category of RE-AIM which focuses on fidelity to best practices for the EBPs or implementation strategies [36, 37]. Two studies [56, 57] compared the average number of smoking-focused sessions delivered to patients in the integrated treatment group to patients in the treatment as usual group. Another study [62] evaluated changes in the number of medication units (e.g., 1 NRT lozenge) and doses (e.g., 7 mg, 14 mg, 21 mg) dispensed after the implementation strategy.

#### Other characteristics

Implementation ranged in length from less than one day [50, 58] to 36 months [53, 57]. Some studies did a combination of implementing smoking bans [55, 62, 63, 65], mandating smoking documentation [51, 61, 65], or developing a new clinical service [61], which went into effect on a specific date and were assumed to persist indefinitely. Five studies representing four implementation programs described their implementation strategies being informed by a theory, model, or framework: one [52] used CFIR [34], two [63, 64] used models of organizational change, and two [53, 59] used a combination of organizational change and social cognition theories.

### Risk of bias

All five RCTs [50, 54, 56, 57, 64] were rated as having some concerns due to lack of detail regarding randomization procedures. Of the 15 non-randomized studies, one was at critical risk of bias due to unclear outcome measurement [66]; 11 [51–53, 55, 58–60, 63, 65, 67, 68] had serious risk of bias due to poor consideration of confounds and absence of a pre-specified data analysis plan; and three [61, 62, 69] had moderate risk of bias due to better accounting for confounding but not having a pre-specified data analysis plan. Most non-randomized studies will be at least at moderate risk of bias due to confounding [39]. The study at critical risk of bias was not included in the data synthesis, in line with guidance from the Cochrane Group [39]. Risk of bias results are summarized in Table 2 and detailed justifications are summarized in supplemental files 2 and 3.

**Table 2:** Risk of bias by study design and study a. Note. D1 = Confounding, D2 = Selection of participants into study, D3 = Classification of intended interventions, D4 = Deviation from intended interventions, D5 = Missing data, D6 = Outcome measure, D7 = Selective reporting,. b. Note. D1* = Randomization - for cluster randomized trial, D1 divided into sub-domain a (randomization) and b (timing of participant recruitment), D2 = Deviation from intended intervention, D3 = Missing data, D4 = Outcome measure, D5 = Selective reporting

non-randomized study	D1	D2	D3	D4	D5	D6	D7	OVERALL
Carillo et al., 2016	moderate	low	low	low	serious	low	moderate	serious
Chen et al., 2018	serious	moderate	serious	no information	low	low	moderate	serious
Correa-Fernández et al., 2019	serious	low	low	low	moderate	serious	serious	serious
Hollen et al., 2010	serious	low	low	low	low	low	low	serious
Huddlestone et al., 2018	serious	low	low	moderate	low	low	serious	serious
Kanter Bax et al., 2020	serious	low	serious	moderate	no information	critical	serious	critical
Lappin et al., 2020	serious	low	low	low	low	low	low	serious
Muladore et al., 2018	serious	moderate	low	low	no information	moderate	moderate	serious
Nitturi et al., 2021	serious	low	low	low	low	moderate	serious	serious
Nitturi et al., 2021 a	moderate	moderate	low	low	moderate	serious	moderate	serious
Okoli et al., 2018	moderate	low	low	low	moderate	low	low	moderate
Parker et al., 2012	serious	serious	serious	low	low	low	serious	serious
Scharf et al., 2011	moderate	low	low	low	low	low	moderate	moderate
Scheeres et al., 2020	serious	low	low	low	low	low	low	serious
Wye et al., 2017	moderate	low	low	low	low	low	moderate	moderate

Randomized controlled trial	D1a*	D1b*	D2	D3	D4	D5	OVERALL
Brunette et al., 2015	Some concerns	N/A	Low	Low	Low	Some concerns	Some concerns
McFall et al., 2005	Some concerns	N/A	Low	Low	Low	Some concerns	Some concerns
McFall et al., 2010	Some concerns	N/A	Some concerns	Low	Low	Low	Some concerns
Dixon et al., 2009	Some concerns	N/A	Some concerns	Some concerns	Low	Some concerns	Some concerns
Schnoll et al., 2023	Some concerns	Some concerns	Low	Low	Low	Low	Some concerns

### Data synthesis

Meta-analysis was not feasible due to heterogeneity in the design and outcomes of studies, which mirrors most prior systematic reviews of implementation strategies to improve delivery of tobacco interventions [26, 28]. In addition to a narrative synthesis, we also provide a quantitative synthesis of absolute increases in the reach of tobacco interventions to smoking patients after implementation strategies, but do not draw conclusions about the strength or clinical significance of these changes given the absence of a meta-analysis. Quantitative outcomes are in Table 3.

**Table 3:** Absolute changes in reach, adoption, and effectiveness by study and statistical significance Note. ^a^Provider adoption of 5As. ^b^Hospital adoption of 5As and tobacco education for providers. ^c^Did not report p-values. Kanter-Bax not included in data synthesis due to being at critical risk of bias. ^d^NRT or bupropion. ^e^Group or individual counseling. ^*f*^*Assist* with referrals to medication or counseling. ^g^70% absolute increase for counseling and 36% absolute increase for NRT or NRT + bupropion. ^±^ statistically significant; ^*^No statistically significant change or no p-value reported. NRT = Nicotine Replacement Therapy; OR = Odds Ratio

	Brunette 2015	Carrillo 2017	Chen 2018	Correa-Fernandez 2019^a^	Dixon 2009	Hollen 2010^b^	Huddle-stone 2018^c^	Lappin 2020	McFall 2005	McFall 2010	Muladore 2018	Nitturi 2021^d^	Nitturi 2021^a^	Okoli 2018	Parker 2012^c^	Scharf 2011	Scheeres 2020	Schnoll 2023	Wye 2017
REACH OR ADOPTION																			
Ask		12^±^		13^±^			−10*	9^±^					13^±^	2^±^					16^±^
Advise				16^±^			−9*						17^±^						8^±^
Assess				18^±^									19^±^						
Assist				31^±^									32^±^						5^±^
Assist with medications		11*^d^	13^±^														5^±^	6^±^	
Assist with NRT	2.94^±^			OR = 3.3^±^	16^±^	12^±^	31*	16^±^			20*			5*	8*			4^±^	18^±^
Assist with non-NRT	−0.61^±^			OR = 1.5^±^	9^±^	32^±^												2^±^	
Assist with counseling^e^		59^±^		OR = 1.2*										16*					
Assist with referrals^f^		53^±g^			11^±^		30*												
Arrange				19^±^									20^±^						
ADOPTION OF PROVIDER TOBACCO EDUCATION				44^±^		11*						35^±^							
EFFECTIVENESS			3% decrease in smoking prevalence^±^		5% decrease in smoking prevalence for whole sample^±^				12% vs. 3% 7-day point prevalence abstinence in integrated care and usual care, respectively*	9% integrated care vs. 5% usual care 12-month prolonged abstinence^±^					7% of inpatients quit and 17% of community patients quit*			4% quit rate in each group*	

#### Reach and adoption

Statistically significant improvements in the reach or adoption of the 5As were present for 100% of studies that *adapted and tailored to context*, 89% of studies that *developed stakeholder interrelationships*, 89% of studies that *provided interactive assistance*, 87% of studies that *trained and educated stakeholders*, 86% of studies that *engaged consumers*, 78% of studies that *utilized financial strategies*, 77% of studies that *changed* infrastructure, 76% of studies that *used evaluative and iterative strategies*, and 70% of studies that *supported clinicians*.

#### Reach by setting

**Fig. 2:**
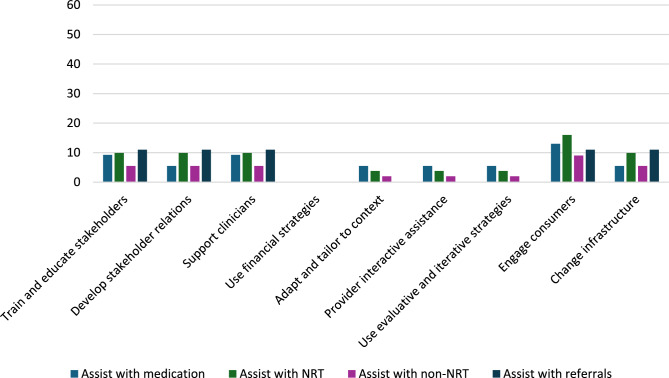
Percent change in reach outcomes by implementation domain for outpatient studies Average change in reach outcomes, reported as percentage of patients receiving 5As, between baseline and follow-up by ERIC implementation domain for n = 3 outpatient studies

**Fig. 3:**
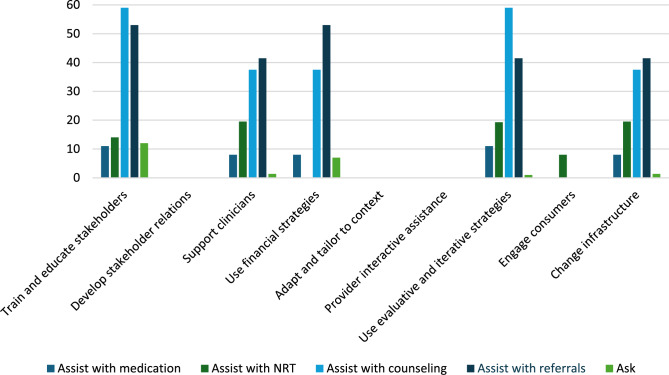
Percent change in reach outcomes by implementation domain for inpatient studies Average change in reach outcomes, reported as percentage of patients receiving 5As, between baseline and follow-up by ERIC implementation domain for n = 5 inpatient studies

Patient-level reach outcomes were reported by 13 studies [50–52, 54, 58, 61, 63–69], one of which was at critical risk of bias and will not be included in data synthesis [66]. Among the 12 eligible reach studies, four evaluated the reach of 5As across all eligible patients during the study period regardless of smoking status [50, 63, 68, 69] and eight [51, 52, 54, 58, 61, 64, 65, 67] evaluated the reach of *Ask* across all eligible patients and the reach of the remaining 4As across only smoking patients. Measuring the reach of tobacco interventions to all patients can be misleading if smoking rates for the sample change over time. Thus, a quantitative synthesis was completed for the eight studies that evaluated the reach of the 4As to smoking patients only, three [52, 54, 64] of which were conducted in outpatient and five [51, 58, 61, 65, 67] of which were conducted in inpatient settings. These eight studies represented 12,106 patients. The two RCTs [54, 64] had some concerns of bias and all six non-randomized studies [51, 52, 58, 61, 65, 67] were at serious risk of bias. Average absolute increases in reach by ERIC domain and setting for these eight studies are illustrated in Figs. 2 and 3.

In the three outpatient studies evaluating reach of the 5As, the most common outcome evaluated was *Assisting* with NRT (*n* = 3). *Asking*, *Advising*, *Assessing*, *Assisting* with counseling, and *Arranging* follow-up were not evaluated in outpatient studies. The greatest absolute increases after, compared to before implementation, were seen for *Assisting* with referrals (11%), followed by *Assisting* with NRT (10%), *Assisting* with any tobacco cessation medication (9%) and *Assisting* with non-NRT (6%).

For the five inpatient studies, *Assisting* with NRT (*n* = 4) was the most common outcome evaluated. *Assessing*, *Assisting* with non-NRT, and *Arranging* follow-up were not evaluated in these studies. The greatest absolute increases were seen for *Assisting* with referrals (42%), followed by *Assisting* with counseling (38%), *Assisting* with NRT (16%), and *Assisting* with any tobacco cessation medication (11%).

#### Reach by setting and strategy domain

##### Adapt and tailor to context

**Fig. 4:**
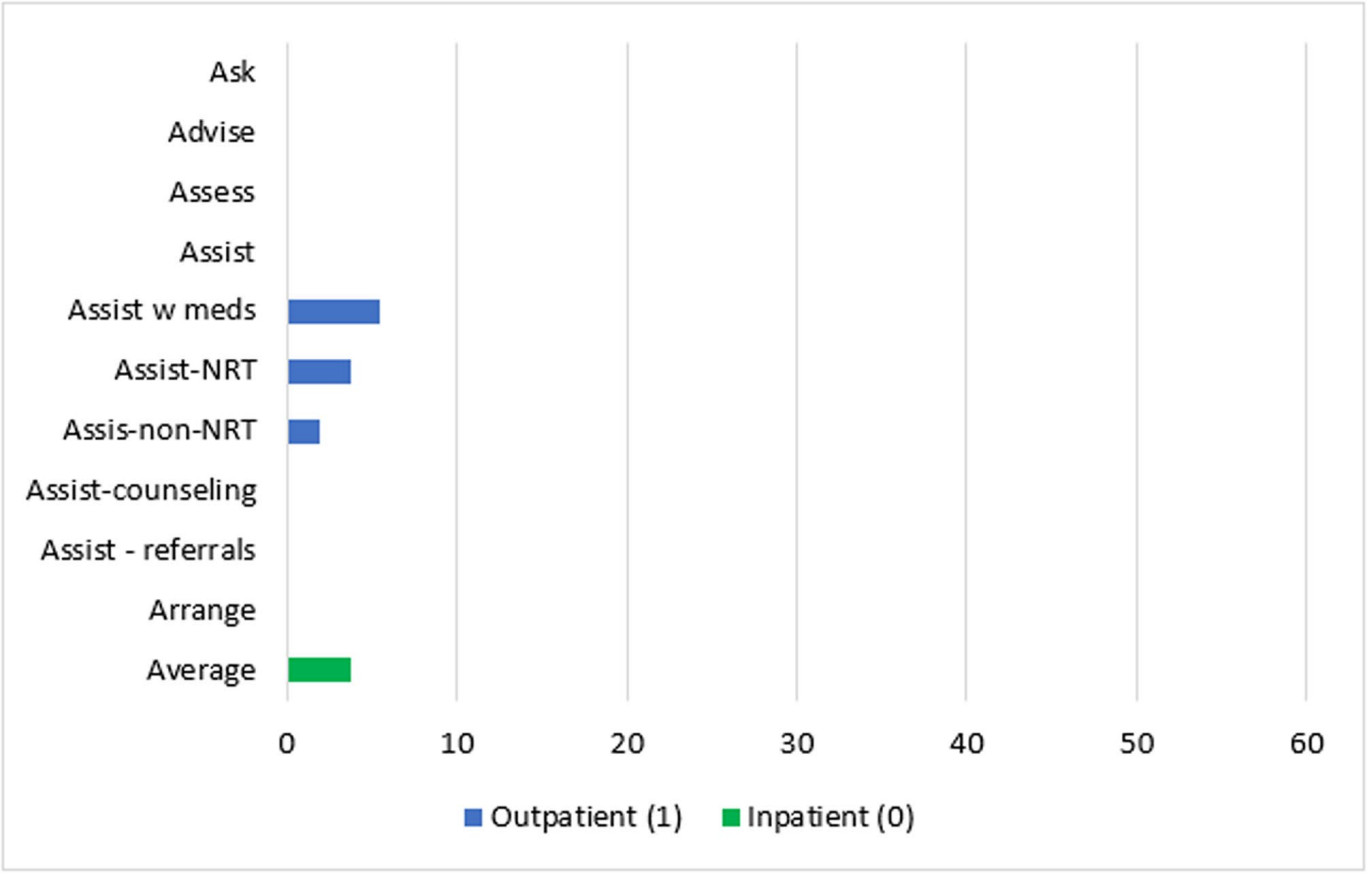
Average absolute increases (%) for studies that *adapt and tailor to context* Average change in reach outcomes, reported as percentage of patients receiving 5As, between baseline and follow-up for n = 1 study that used strategies from ERIC domain: *adapt and tailor to context*

The study [64] that used strategies in the *adapt and tailor to context* ERIC domain was conducted in an outpatient setting and reported increases in reach outcomes at the sites with the experimental Addressing Tobacco Through Organizational Change (ATTOC) implementation program: 6% increase for *Assisting* with any tobacco medication, 4% increase for *Assisting* with NRT, and 2% increase for *Assisting* with NRT (Fig. 4).

##### Train and educate stakeholders

**Fig. 5:**
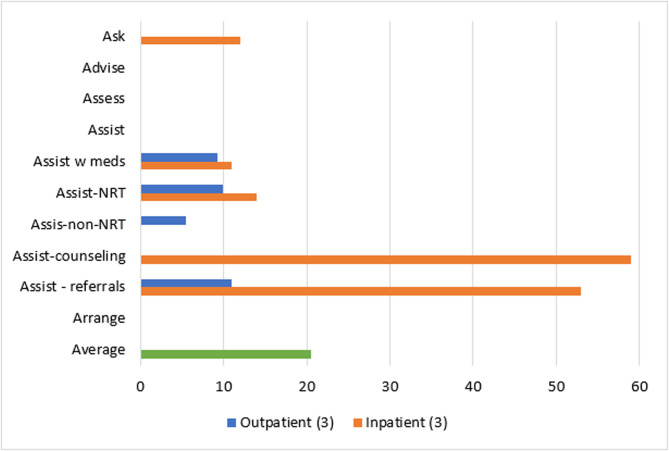
Average absolute increases (%) for studies that *train and educate stakeholders* Average change in reach outcomes, reported as percentage of patients receiving 5As, between baseline and follow-up for n = 6 studies that used strategies from ERIC domain:* train and educate stakeholders*

Three outpatient [52, 54, 64] and three inpatient [51, 58, 67] studies used strategies in the *train and educate stakeholders* domain. Improvements in *Ask* were only evaluated in inpatient studies at an average absolute increase of 12% after implementation. *Advise* and *Assess* were not evaluated in studies using strategies from the *train and educate stakeholders* domain. *Assisting* with any medications was similar for outpatient and inpatient studies (9% vs. 11% increase) and *Assisting* with NRT was slightly lower for outpatient compared to inpatient studies (10% vs. 14% increase). Changes in *Assisting* with non-NRT were evaluated in one outpatient study at an absolute increase of 6%. *Assisting* with counseling in this domain produced a 59% increase across inpatient studies and *Assisting* with referrals increased by 11% for outpatient and 53% for inpatient studies. *Arranging* follow-ups was not evaluated by studies in this domain. In sum, the greatest increases produced by outpatient studies were for *Assisting* with referrals (11%) and greatest increases for inpatient studies were for *Assisting* with counseling (59%) (Fig. 5).

##### Provide interactive assistance

**Fig. 6:**
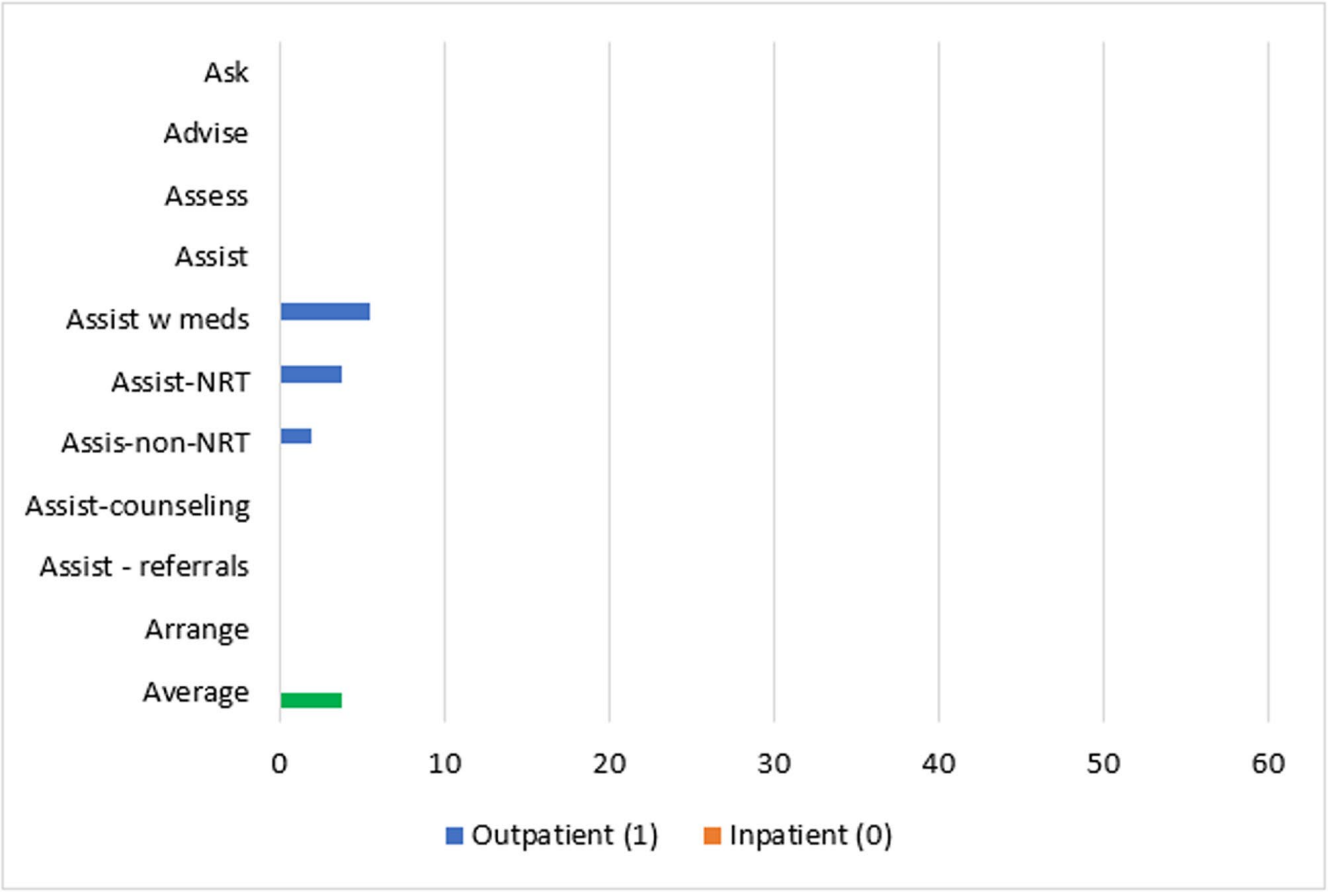
Average absolute increases (%) for studies that *provide interactive assistance* Average change in reach outcomes, reported as percentage of patients receiving 5As, between baseline and follow-up for n = 1 study that used strategies from ERIC domain: *provide interactive assistance*

The same outpatient study that used strategies in the *adapt and tailor to context* domain also used strategies in the *provide interactive assistance* domain [64] (Fig. 6).

##### Develop stakeholder interrelationships

**Fig. 7:**
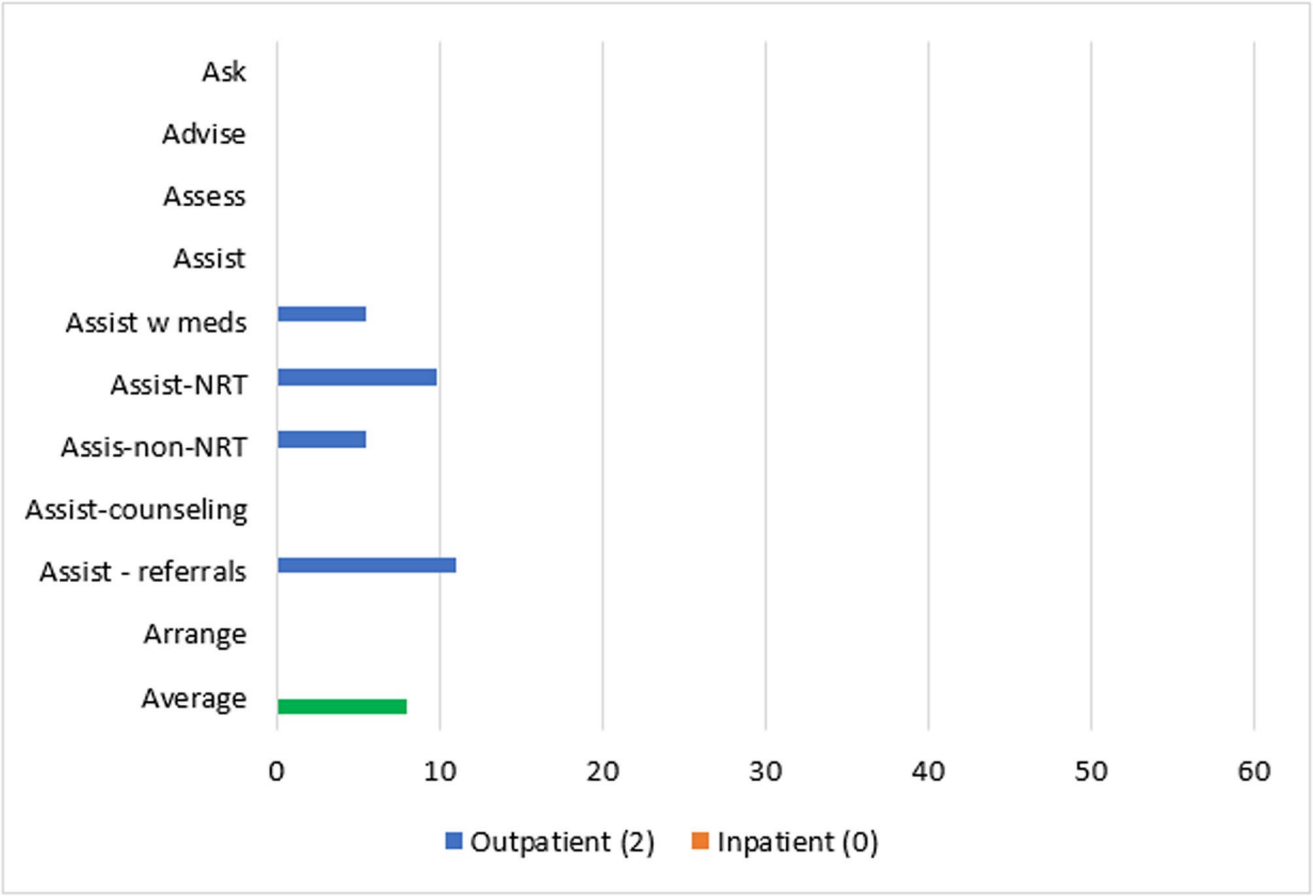
Average absolute increases (%) for studies that *develop stakeholder relations* Average change in 5As reach outcomes, reported as percentage of patients receiving 5As, between baseline and follow-up for n = 2 studies that used strategies from ERIC domain: *develop stakeholder relations*

Two outpatient studies [54, 64] *developed stakeholder interrelationships* for average absolute increases of 6% for *Assisting* with any tobacco medication, 10% for *Assisting* with NRT only, 6% for *Assisting* with non-NRT, and 11% for *Assisting* with referrals (Fig. 7).

##### Engage consumers

**Fig. 8:**
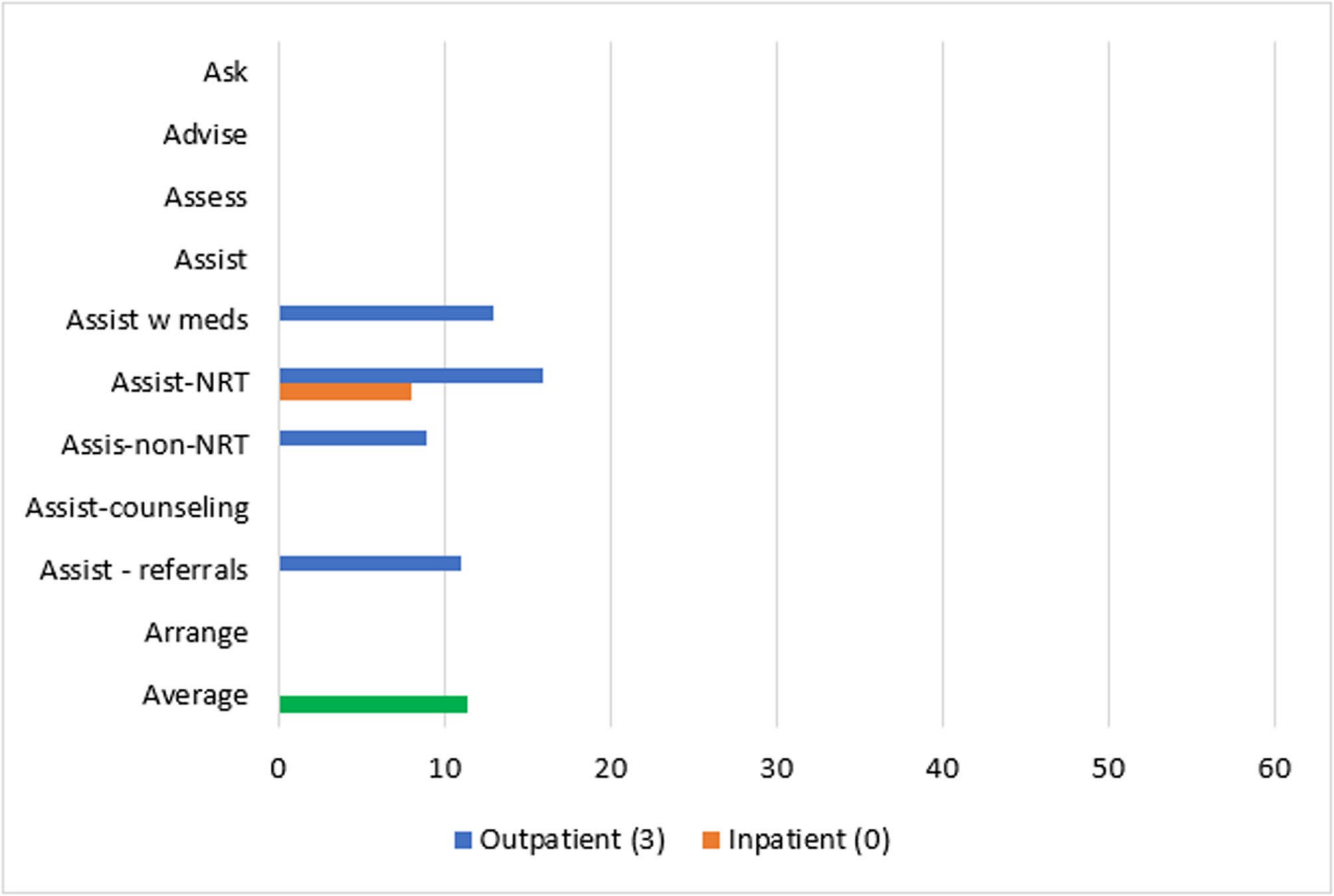
Average absolute increases (%) for studies that *engage consumers* Average change in reach outcomes, reported as percentage of patients receiving 5As, between baseline and follow-up for n = 3 studies that used strategies from ERIC domain: *engage consumers*

Two outpatient studies [52, 54] and one inpatient study [67] *engaged consumers.* For outpatient studies, average absolute increases after implementation were 13% for *Assisting* with tobacco medication, 9% for Assisting with non-NRT, and 11% for *Assisting* with referrals. Contrary to the pattern for other ERIC domains, outpatient studies that engaged consumers had much greater increases in *Assisting* with NRT compared to the inpatient study (16% vs. 8%) (Fig. 8).

##### Utilize financial strategies

**Fig. 9:**
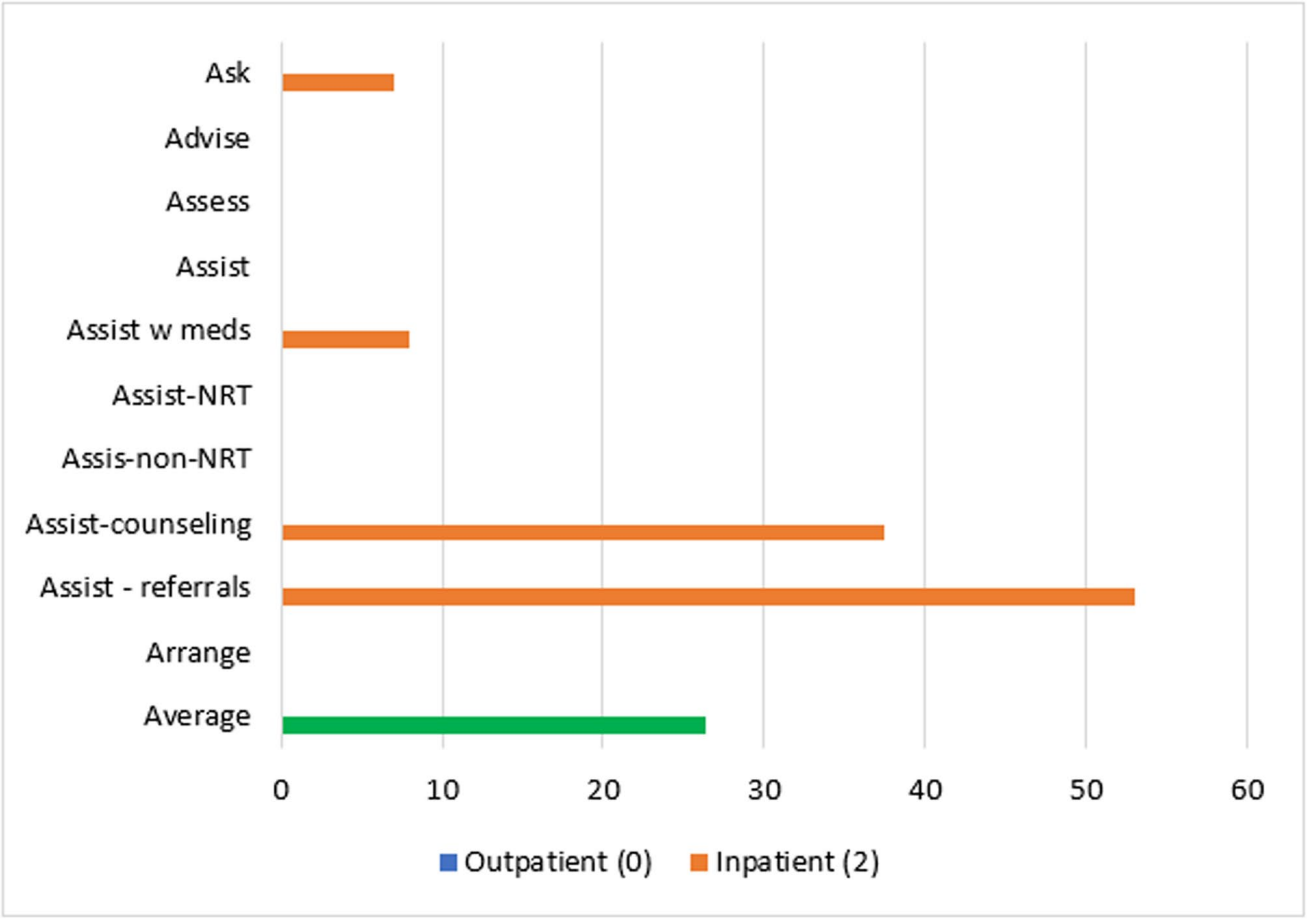
Average absolute increases (%) for studies that *use financial strategies* Average change in reach outcomes, reported as percentage of patients receiving 5As, between baseline and follow-up for n = 2 studies that used strategies from ERIC domain: *use financial strategies*

Two inpatient [51, 61] studies *utilized financial strategies* and produced average absolute increases, after implementation, of 7% for *Asking* about tobacco use, 8% for *Assisting* with tobacco medications, 38% for *Assisting* with counseling, and 53% for *Assisting* with referrals (Fig. 9).

##### Use evaluative and iterative strategies

**Fig. 10:**
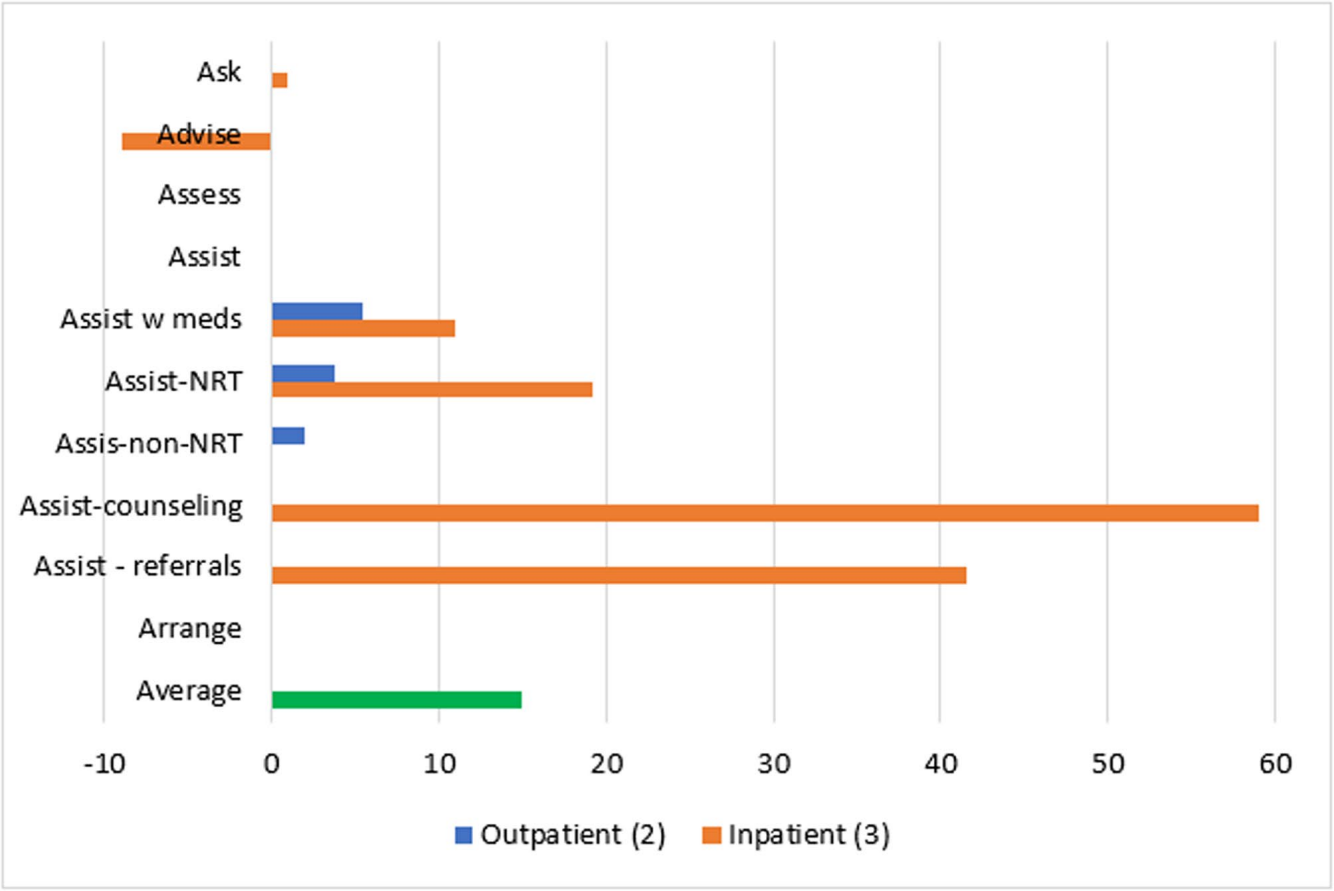
Average absolute increases (%) for studies that *use evaluative and iterative strategies* Average change in reach outcomes, reported as percentage of patients receiving 5As, between baseline and follow-up for n = 5 studies that used strategies from ERIC domain: *use evaluative and iterative strategies*

Two outpatient [52, 64] and three inpatient [51, 65, 67] studies *used evaluative and iterative strategies*. *Asking* about tobacco use and *Advising* tobacco cessation were evaluated in two inpatient studies and involved a 1% increase and 9% decrease, respectively. Average increases for *Assisting* with medication and *Assisting* with NRT were lower for outpatient than inpatient studies after implementation: 6% versus 11% for any tobacco medication and 4% versus 19% for NRT. Assisting with non-NRT was only evaluated in outpatient studies at an average increase of 2%. *Assisting* with counseling and with referrals were only evaluated in inpatient studies, with increases of 59% and 42%, respectively (Fig. 10).

##### Change infrastructure

**Fig. 11:**
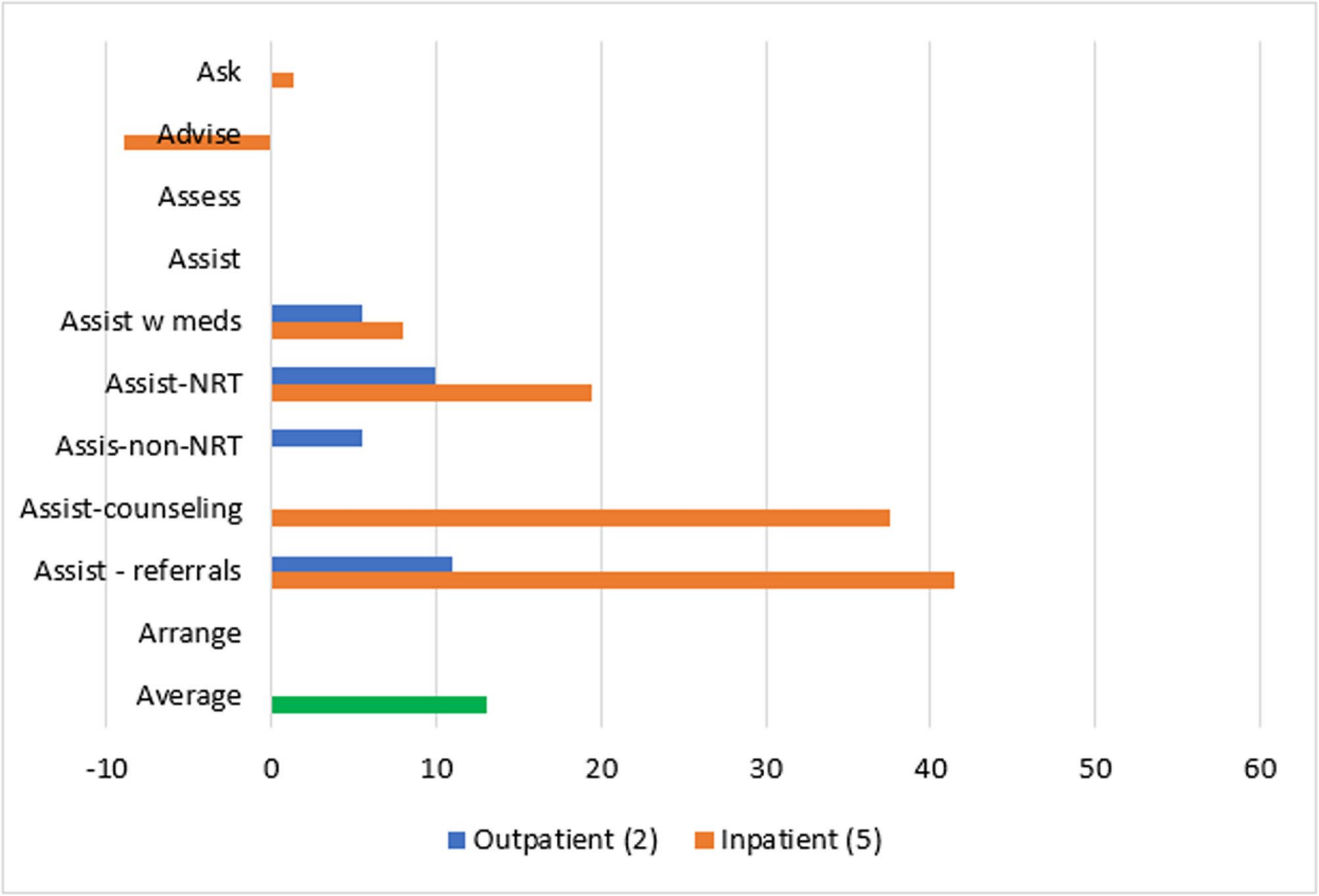
Average absolute increases (%) for studies that *change infrastructure* Average change in reach outcomes, reported as percentage of patients receiving 5As, between baseline and follow-up for n = 7 studies that used strategies from ERIC domain: *change infrastructure*

The domain of *changing infrastructure* was evaluated in two outpatient [54, 64] and five inpatient [51, 58, 61, 65, 67] studies. Inpatient studies produced a 1% absolute increase in *Asking* about Tobacco Use and 9% decrease in *Advising* quit after implementation. Outpatient and inpatient studies that used strategies to *change infrastructure* reported similar changes in *Assisting* with any tobacco medication (6% increase for outpatient and 8% increase for inpatient), and absolute increases in *Assisting* with NRT were smaller for outpatient compared to inpatient studies (10% vs. 20%). Only outpatient studies evaluated *Assisting* with non-NRT and produced an absolute increase of 6%. Only inpatient studies evaluated *Assisting* with counseling and produced an average increase of 38%. Increases were much lower for *Assisting* with referrals for outpatient versus inpatient studies (11% versus 42%) (Fig. 11).

##### Support clinicians

**Fig. 12:**
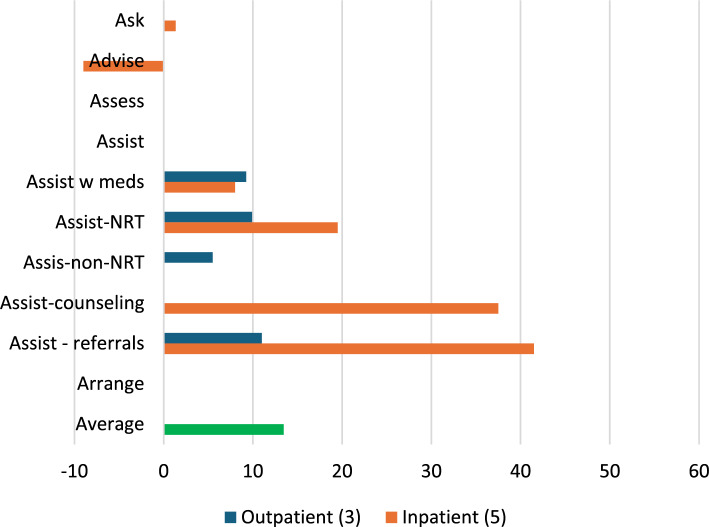
Average absolute increases (%) for studies that *support clinicians* Average change in reach outcomes, reported as percentage of patients receiving 5As, between baseline and follow-up for n = 8 studies that used strategies from ERIC domain: *support clinicians*

Implementation strategies that *supported clinicians* were evaluated in three outpatient [52, 54, 64] and five inpatient [51, 58, 61, 65, 67] studies. Only inpatient studies evaluated *Asking* about Tobacco Use at an average absolute increase of 1% and decrease of 9%, respectively, after implementation. Changes in *Assisting* with any tobacco medication were similar for both outpatient and inpatient settings (9% versus 8% increase), but smaller in outpatient settings for *Assisting* with NRT (10% versus 20% increase). Average absolute increases were 6% for the outpatient studies that evaluated *Assisting* with non-NRT, 38% for the inpatient studies evaluating *Assisting* with counseling, and 11% for outpatient studies evaluating *Assisting* with referrals and 42% for inpatient studies evaluating *Assisting* with referrals (Fig. 12).

#### Adoption

One study [64] evaluated the frequency by which providers reported delivering the 5As on an ordinal level (0 = never to 5 = always) and found a statistically significant increase in staff-reported delivery of any 5A tobacco interventions over time (β = 4.77, 95% CI:3.83–5.70, *p* <.001) at both experimental sites using the ATTOC implementation program and the sites in the training only condition; there were no significant differences between conditions over time. This study was an RCT with some bias concerns.

Two outpatient studies [53, 59] using the same TTTF implementation program evaluated changes in provider adoption of the 5As and found statistically significant changes after implementation in *Asking* about tobacco use (13% and 13% increase), *Advising* quitting (16% and 17% increase), *Assessing* motivation to quit (18% and 19% increase), *Assisting* with any type of tobacco intervention (31% and 32% increase), and *Arranging* follow-up (19% and 20% increase). The Correa-Fernández study [53] reported statistically significant greater odds of delivering NRT (OR = 3.29, *p* <.001) and non-NRT (OR = 1.52, *p* =.04) after the implementation strategy; there was also an increase in *Assisting* with counseling that was not statistically significant (OR = 1.15, *p* =.28). Both studies were at serious risk of bias.

One study [55] at serious risk of bias evaluated changes in the proportion of state psychiatric hospitals *Assisting* with tobacco medication that adopted a smoke-free policy (*change infrastructure*) and implemented trainings (*train and educate stakeholders)*. This study found that hospitals that adopted a smoke-free policy did not show statistically significant absolute increases in offering NRT (12%, *ps* > 0.05) but did produce significant increases in offering non-NRT after implementation (absolute increase 32%, *p* <.05) [55]. Hospitals that still allowed smoking did not demonstrate a statistically significant change in NRT or other pharmacotherapy offered after smoke-free policy implementation [55].

Two outpatient [53, 60] and one inpatient [55] study evaluated changes in provider adoption of educational programs designed to support increased delivery of tobacco interventions. Correa-Fernández [53] used the TTTF implementation program that covered six ERIC domains (*use evaluative and iterative strategies*,* provide interactive assistance*,* develop stakeholder interrelations*,* train and educate stakeholders*,* engage consumers*, and *change infrastructure*); this study reported 35–53% absolute increases and increased odds (OR range = 5.36–18.89, *p*s < 0.0001) of provider exposure to various educational seminars on tobacco use and interventions. Nitturi [60] used *evaluative and iterative strategies*,* developed stakeholder relations*, and *trained and educated stakeholders* and increased the number of tobacco-focused educational programs offered to providers by 35%. The inpatient study [55] reported an 11% average increase (range = 7% − 18%) in provider participation in tobacco cessation educational programs that was not statistically significant after hospitals implemented strategies in the *training and educating stakeholders* and *change infrastructure* domains; in this study, hospitals that did not use these strategies experienced a statistically significant decline in number of tobacco-related training offered to providers. All three studies were at serious risk of bias.

#### Secondary outcomes

##### Implementation

Two outpatient studies by McFall [56, 57] that used strategies in the *train and educate stakeholders* and *support clinicians* domains reported that patients working with clinicians trained to integrate smoking cessation and PTSD treatment had 2–7 more smoking cessation counseling sessions than those referred to a separate smoking clinic. An inpatient study by Scharf [62] evaluated changes in NRT units (unit = 1 patch, 1 piece of gum, 1 lozenge) and doses dispensed before and after an implementation strategy that *trained and educated stakeholders*,* supported clinicians*, and *changed infrastructure* and found that average monthly NRT units significantly increased at 3-year follow-up (254 to 4,468 units, F [1, 11] = 67.76, *p* <.001). Specifically, there were greater increases for gum or lozenges than patches (F [1, 22] = 26.62, *p* <.0001) and use of high-dose patches increased more than low-dose patches over time F [1, 22] = 736.62, *p* <.0001), with more 21 mg than 7 mg patches prescribed (*p* <.0001) [62].

##### Effectiveness

Five outpatient studies and one inpatient study examined changes in smoking behavior after implementation efforts in the form of changes in quit rates [56, 57, 64, 66, 67], quit odds [57], or smoking prevalence [52, 54]. Chen [52] reported a 3% statistically significant decrease in smoking prevalence. McFall 2005 [56] did not find a statistically significant difference in 7-day point prevalence abstinence between integrated care and usual care (12% vs. 3%), and McFall 2010 [57] found that with integrated care Veterans had increased odds of 12-month prolonged abstinence compared to usual care (OR = 2.26, 95% CI, 1.30–3.91; *p* =.004). Dixon [54] found a 5% decrease in smoking prevalence, but no difference in smoking prevalence between immediate and delayed implementation sites. Parker [67] reported a 7% quit rate for inpatients and 17% quit rate for outpatients, but did not report statistical tests. Schnoll [64] found no difference in quit rates between sites that received ATTOC (4% quit rate) and sites that received provider training only (4% quit rate). The four RCTs [54, 56, 57, 64] had some concerns of bias and all non-randomized studies [52, 67] were at serious risk of bias.

## Discussion

Individuals with mental illness smoke at double the rate of those without mental illness [4] and most want to quit [2, 9, 10]. A plethora of evidence-based tobacco treatment interventions can support smoking reductions in this population, but mental health providers are hesitant to deliver such interventions. Increasing mental health provider adoption of tobacco treatment is necessary to expand its reach to these patients and reduce smoking-related health inequities between those with and without mental illness. The literature evaluating implementation strategies designed to improve delivery of tobacco interventions in mental health settings is nascent and promising. Most implementation efforts included a combination of educating providers, changing the environment to support tobacco intervention delivery, establishing system-level rules to guide smoking interventions, and reducing barriers to smoking intervention delivery. The combination of these strategies was associated with increased tobacco intervention delivery in both inpatient and outpatient mental health settings. At the same time, due to high risk of bias and heterogeneity in study outcome metrics, this summary should be interpreted with caution.

### Implementation domain by setting

Commonalities in the types of implementation strategies used in outpatient and inpatient mental health settings can provide insight into barriers to delivering tobacco interventions shared by these settings. Strategies from the *train and educate stakeholders* and *support clinicians* domains were among the three most frequent implementation strategies in outpatient and inpatient settings. Strategies in these domains such as *develop educational materials*, *conduct ongoing training*, *relay data to providers*, and *remind clinicians* may be particularly important to address common barriers to delivering tobacco interventions in mental health settings, such as little knowledge of available tobacco interevntions and competing clinical priorities [17–19]. Of note, a review of implementation trials within health care settings found that strategies in the *train and educate stakeholders* domain were among the most frequently-evaluated strategies, but strategies from the *supporting clinicians* domain were not frequently reported [70]. It is possible that *supporting clinicians* strategies may have special utility for mental health settings, the nature of tobacco interventions, or both.

Some implementation strategies differed across settings. *Changing infrastructure* was more common in inpatient (100% of studies) than outpatient settings (44% of studies). The inpatient milieu is, itself, a therapeutic intervention that must be flexible in response to changing social and public health demands [45]. Thus, changes to the milieu can be made sweepingly and rapidly. Though changing infrastructure can still be implemented in outpatient settings, such strategies may be more feasible and acceptable to providers in inpatient, relative to outpatient, settings. *Mandate change*, another strategy in the *changing infrastructure* domain, in the form of smoking restrictions was a common strategy used by inpatient settings in this domain and has important historical context. The four inpatient studies that implemented smoking bans did so in 2007, 2015, 2016, and 2018. It is significant, yet not surprising, that many inpatient psychiatric settings have maintained complicit smoking policies for nearly 20 years after the tobacco industry partnered with mental health advocacy groups in the US to successfully lobby for exemption of psychiatric units from the 1990 Joint Commission on Accreditation of Healthcare Organizations (JCAHO) smoking restrictions [71]. Smoking ban exemptions in inpatient psychiatric facilities also persisted in many Australian jurisdictions until 2015, when all exemptions were revoked [72]. The narrative that the benefits of smoking outweigh the consequences for individuals with mental illness remains deeply entrenched, is reflected in prolonged efforts to delay evidence-based smoking policies in mental health settings, and contributes to mental health provider hesitance to treat smoking in their patients [24].

Strategies in the *use evaluative and iterative strategies* domain, particularly *audit performance and provide feedback*, *obtain and use patient and family feedback*, and *assess for readiness and identify barriers* were used by more outpatient than inpatient studies (67% versus 50%). It may be easier to obtain feedback from patients in an outpatient setting given the transience of hospitalized patients. However, auditing performance with feedback has a strong evidence base for improving health care practices and would be beneficial in any health care setting considering an implementation program [70, 73, 74]. Additionally, identifying barriers is a key component to developing an effective implementation program [34]. It is worth exploring how these evaluative strategies can be more frequently implemented in inpatient settings and whether using these strategies in inpatient settings can improve tobacco intervention delivery.

### Outcome by setting

Change in reach of 5As to patients (i.e., proportion of patients who received 5As) was the most common outcome assessed across both inpatient and outpatient settings. In outpatient studies, *Assisting* with referrals also produced the largest average increases (11%), followed closely by *Assisting* with NRT (10%). The smallest change in outpatient studies was for *Assisting* with non-NRT (6%). Among inpatient studies, the greatest average absolute increases were for *Assisting* with referrals (42%) and *Assisting* with counseling (38%), and there was an average 9% decrease in *Advising* quit. Two [51, 65] of the three studies that evaluated *Assisting* with referrals were conducted in inpatient settings, where referrals to outpatient services are necessary for care continuity and also consistent with best practices for sustaining quit attempts after discharge [75]. In the outpatient study [54], psychiatrists were the focus of the implementation program and were asked about referring to outpatient group counseling – psychiatrists and patients alike may have welcomed the opportunity for additional follow-up on smoking if there are delays of several months between psychiatry appointments.

Although referring patients to tobacco treatment may be easier for outpatient mental health providers and increase the intensity of tobacco interventions, patients may encounter barriers during the referral process. For example, tobacco interventions could be delayed if there is a waitlist or a lengthy referral process, or mental health providers could talk about smoking less if they believe making a referral means they no longer have to follow-up about smoking. Clinical practice guidelines for tobacco interventions state that that physicians should follow-up about smoking with patients during every follow-up visit, and the World Health Organization recommends that tobacco interventions be integrated into routine mental health care to reduce access barriers for patients [8, 75]. Therefore, mental health clinics should evaluate their local barriers and facilitators to tobacco intervention delivery, evaluate potential benefits and consequences of establishing speciality smoking cessation programs or referring patients to external resources, and create decision aids to help providers determine when it is appropriate to outsource tobacco interventions, particularly if the referring provider already has the credentials to prescribe medications or deliver brief behavioral counseling.

Few studies evaluated changes in varenicline (formerly Chantix) and bupropion prescriptions. In 2008, the US FDA issued a black box warning for Chantix and bupropion in response to anecdotal reports of neuropsychiatric adverse events [13]. This was accompanied by a decline in prescribing, including for those with mental illness [22, 76, 77]. After a large randomized trial and VA pharmacovigilance trial re-establishing varenicline’s safety and effectiveness, including for those with serious mental illnesses like schizophrenia, the black box warning was removed in 2016 [13, 14]. However, prescribing levels have yet to return to their pre-black box warning peak [76].

Notably, none of the inpatient studies evaluated changes in non-NRT medications. There are a few potential reasons for this. First, most inpatient studies overlapped with the black box warning. Thus, many prescribers were likely hesitant to prescribe these medications to psychiatric inpatients. Second, providers may view it as unnecessary to prescribe a non-NRT medication when a patient’s smoking behavior has lessened or stopped during a hospitalization. Relatedly, bupropion and Chantix involve escalating and then titrating doses around a quit date, which can be challenging to do during a short inpatient stay and sustain after discharge, especially if there are barriers to care coordination between inpatient and outpatient prescribers. At the same time, use of bupropion and Chantix up to six months after quitting helps sustain quit attempts for patients with mental illness [78]. Thus, these medications must be seriously considered as treatment options for all patients interested in maintaining quit after discharge from a psychiatric hospital.

Among the outpatient studies, prescribing non-NRT had the smallest absolute increase compared to other types of tobacco interventions. Given that medications such as bupropion and Chantix produce superior quit rates for people who smoke, including those with mental illness, there may be major barriers to improving delivery of these medications in mental health settings [13, 15]. Overlap with the FDA black box warning period may partially explain low prescription rates and subtle changes over time. However, prescribing trends have remained low and yet to return to peak levels even after removal of the black box warnings [76, 77]. These warnings may be continuing to impact prescriber willingness to deliver these medications to people with mental illness who smoke, which could make quit attempts for this population more challenging [13, 76, 77]. Providers may be more likely to increase provision of counseling or referrals than tobacco cessation medication if they perceive counseling as less potentially harmful [79]. System-level factors like formulary restrictions and limited access to prescribers could also be contributing to these modest non-NRT prescribing increases [17]. Exploring clinic-level barriers to tobacco cessation medication prescribing and tailoring strategies accordingly will be essential in efforts to improve prescribing of all FDA-approved tobacco cessation medication in mental health settings.

### Implementation strategy by setting and outcome

Overall, most implementation programs improved reach of tobacco interventions to patients and provider adoption of tobacco education across a variety of mental health settings, but the average change in reach varied by implementation strategy domain and setting. As stated previously, only eight studies could be included in a quantitative synthesis of reach of *Advising*,* Assessing*,* Assisting*, and *Arranging* to patients who smoke. For outpatient studies, the greatest increases in *Assisting* with medication (13%), *Assisting* with NRT (16%), and *Assisting* with non-NRT (9%) were for studies that *engaged consumers*. *Assisting* with referrals was equally effective (11% absolute increase) for studies that used strategies from the domains of *train and educate stakeholders*,* develop stakeholder relations*,* support clinicians*,* engage consumers*,* and change infrastructure.* Within this *engage consumers* domain, most of the outpatient studies used the strategy *prepare patients to be active participants* in the form of educational outreach. One implementation program, TTTF, *used mass media* to disseminate tobacco education to patients [41, 53, 59] and another [66] *involved patients in the implementation process*, though this study was at critical risk of bias and not included in the data synthesis. Providers may face more barriers to prescribing tobacco medication than referring a patient to a tobacco treatment program, and patient engagement may play a major role in addressing these barriers for providers.

For inpatient studies, the greatest increases in *Asking* occurred for studies that *trained and educated stakeholders* (12% increase); improvements in *Assisting* with any tobacco medication were greatest for studies that *trained and educated stakeholders* and *used evaluative and interactive strategies* (11%); *Assisting* with NRT was greatest for studies that *supported clinicians* and *changed infrastructure)*(20%); *Assisting* with counseling was greatest for studies that *trained and educated stakeholders* and *used evaluative and iterative strategies* (59%); and *Assisting* with referrals was greatest for studies that *trained and educated stakeholders* and *used financial strategies* (53%).

Few comparisons could be made between reach outcomes by setting and implementation domain because *Assisting* with any tobacco medication, *Assisting* with NRT, and *Assisting* with referrals were the only outcomes evaluated by at least one outpatient and one inpatient study. But the available comparisons shed light on setting-specific differences in smoking cessation salience (mandated abstinence in inpatient settings), treatment objectives (acute stabilization while hospitalized vs. long-term treatment in the community), and treatment barriers. These setting-specific factors likely made some domains, like *engaging consumers*, more feasible to implement in outpatient settings and other strategies, like *changing infrastructure*, more feasible to implement in inpatient settings. Though only one study [64] explicitly described tailoring strategies to local context, the alignment between strategy type, treatment setting considerations, and favorable tobacco intervention outcomes suggests tailoring may have been occurring much more frequently.

In line with other systematic reviews of implementation efforts in health care settings, several implementation strategies were often delivered simultaneously, making it challenging to link individual strategies with specific tobacco intervention outcomes [70]. However, disentangling the individual strategy effects on tobacco intervention changes may be futile if evaluation efforts do not consider that mental health setting may moderate the relationship between implementation domains and tobacco intervention outcomes. For example, in this review, *Assisting* with referrals improved by 11% in outpatient settings and 42–53% in inpatient settings, with no implementation domain emerging as superior to another in either setting. This suggests that there may be more natural facilitators in inpatient settings that support robust improvements in *Assisting* with referrals to tobacco treatment programs regardless of implementation strategies that are introduced. It is also possible that strategies, as defined by ERIC, may matter less than the change mechanisms (i.e., mediators) promoted by these strategies. For example, Lappin [68] and Wye [69] were conducted in different inpatient settings and used different ERIC strategies, but each addressed all six COM-B behavior change elements (Table 1b). Lappin [68] used six strategies across five ERIC domains and Wye [69] used 11 strategies across seven domains. Both studies also produced statistically significant improvements in *Asking* (9% and 16%, respectively) and *Assisting* with NRT (16% and 18%, respectively) (Table 1).

The combination of strategies used to target behavior change mechanisms may matter more than the quantity of strategies. For example, an implementation evaluation of strategies to improve hepatitis C (HCV) treatment delivery in the Veterans Affairs Health Care System found that 10 strategies across seven of the nine ERIC domains, implemented in five different configurations, distinguished between high and low-performing sites; and each of these configurations, such as only *providing technical assistance* (*provide interactive assistance* domain), or using a combination of *creating new clinical teams*,* sharing quality improvement knowledge*, and *engaging consumers* (from *support clinicians*,* develop stakeholder interrelations*, and *engage consumer* domains), were similarly effective at increasing HCV treatment [80]. At the same time, high-performing sites also systematically differed from low-performing sites such that high-performing sites were more complex in regards to patient volume, patient risk scores, clinical programs, research funds, and training programs [80]. Thus, the interaction between the setting in which implementation strategies were embedded may have interacted with the strategies themselves to improve clinical practice.

### Implementation mechanisms by setting

It is also possible that some mechanisms are better to target than others to improve tobacco intervention in mental health settings, and that certain settings are better suited to address specific types of behavior change mechanisms. All inpatient studies targeted opportunity behavior change mechanisms, but all outpatient studies targeted capability mechanisms. As an example, the implementation program used by Huddlestone [65], showed the greatest improvements, compared to other inpatient implementation programs, in providers *Assisting* with NRT (31%) even though it only used three strategies (in the *use evaluative and iterative strategies*, *change infrastructure*,* support clinicians* domains), that targeted four of the six behavior change mechanisms (reflective and automatic motivation, and physical and social opportunity) [33]. Addressing provider opportunities to deliver tobacco interventions may be more feasible and acceptable to providers in inpatient settings, where the milieu functions as a therapeutic intervention [45]. Thus, setting-specific barriers and facilitators may make certain behavior change mechanisms more relevant and the strategies that activate these mechanisms more impactful when deployed in such settings.

In most studies, regardless of setting, the combination of implementation strategies covered all six behavioral domains of the COM-B model [33]. Thus, limited improvements may be related to poor tailoring to local needs or an insufficient dose (i.e., amount of time per provider spent in implementation activities) than a failure to address important components of behavior change (e.g., capability, opportunity, and motivation). For instance, a skills-based educational session (*train and educate stakeholders* ERIC domain, *education* BCW strategy that addresses psychological capability and reflective motivation) about the safety and efficacy of varenicline that is poorly attended or only offered once may not improve varenicline prescribing rates even if it is combined with changes to prescription order sets that remove varenicline prescribing restrictions in the medical record (*change infrastructure* ERIC domain, *environmental restructuring* BCW strategy that addresses automatic motivation, physical opportunity, and social opportunity). Of note, the amount of time spent in implementation activities per provider is associated with more pronounced changes in clinical practice [49, 81]. Although time spent in implementation activities was not documented in included studies, incorporating this variable in future implementation evaluations that focus on tobacco intervention practices can have multiple benefits. First, it could inspire researchers to prioritize delivering strategies in a way that maximizes provider engagement early in the implementation process. Second, it can guide future recommendations about implementation dose per setting or per provider to optimize provider behavior change outcomes.

### Limitations

This systematic review had some limitations. First, we were not able to compare absolute increases in reach of the 5As across all studies due to heterogeneity in study outcomes. Two implementation programs evaluated changes in provider adoption of evidence-based tobacco interevntions, but results were not comparable to one another because one program evaluated changes in the proportion of providers delivering these interventions [53, 59] and the other measured the frequency of treatment delivered using an ordinal scale [64]. Both implementation programs were conducted in outpatient settings. Although patient-level metrics like reach are important, they do not elucidate the frequency and intensity by which providers are changing their behavior. Reach alone does not indicate whether increases are attributed to changes in intervention delivery across all or a select few providers. If changes are only made by a few providers, any intervention improvements may not be robust to common challenges like staff turnover. Measuring provider adoption of tobacco interventions during an implementation evaluation can provide useful information about the acceptability and feasibility of both the implementation strategy and intervention being implemented; and this data can be used to maximize the sustainability of intervention improvements.

Second, most included studies were at serious risk of bias and non-randomized. Thus, we cannot draw causal conclusions about the relationship between implementation strategies and changes in tobacco interventions. Third, we did not evaluate evidence quality. Fourth, rigor could have been enhanced with an additional reviewer for risk of bias evaluations. Finally, we did not conduct a meta-analysis due to heterogeneity in study outcomes and design, which prevented us from drawing conclusions about the strength and certainty of evidence. Evidence quality evaluations and meta-analyses that include moderators and mediators of change are needed, together, to guide recommendations for improving tobacco intervention delivery in mental health settings.

### Recommendations for future implementation research

To maximize the public health impact of tobacco-related clinical practice changes, evaluations of the reach and adoption of the gold-standard smoking interventions, counseling plus medication, are needed [2, 12]. Only one implementation program (TTTF) included in this review evaluated changes in both medication and counseling delivery [64]. It is also essential that future research on this topic monitor changes in prescribing all types of quit medication (i.e., NRT and non-NRT options) given the safety and efficacy of non-NRT options medications like Wellbutrin and varenicline for promoting and sustaining tobacco abstinence in psychiatric populations [12, 13, 15, 82].

To prepare clinicians to deliver gold-standard tobacco interventions, implementation strategies must target all of the 5As. Provider adoption of *Assessing* interest in quitting – but not patient reach of *Assessing* – was only evaluated in one implementation program [53, 59], but is essential to tailoring appropriate tobacco interventions: For example, someone ready to quit smoking within 30 days should be provided appropriate medication and tobacco counseling, but someone who is contemplating quitting should be provided motivational enhancement in the form of the 5Rs [12]. It is reasonable to assume that the absence of this outcome indicates that *Assessment* was not a practice target for providers or implementation strategies. If this was the case, the modest improvements in 5A delivery observed in this review, particularly *Assisting* with medications, could be attributed to providers having insufficient data to guide next steps for tobacco interventions. *Arranging* follow-up was also missing from studies included in this review and could be difficult to do if providers are uncertain about their patient’s interest in quitting. All reach data from studies included in this review were obtained from medical record or pharmacy data; the absence of documentation related to *Assessing* motivation and *Arranging* follow-up in systems where data is obtained for research and quality improvement purposes could be a major barrier to monitoring provider engagement in these tobacco intervention components.

Future research should also consider how to feasibly evaluate quit rates and other changes in smoking behavior over time, which were rarely evaluated in studies included in this review. In prior implementation-focused reviews of tobacco interventions in primary care, approximately half of included studies evaluated quitting or quit attempts [27, 28]. Therefore, the effectiveness of implementation strategies on patient smoking behaviour is unclear based on available data. Of note, the one study included in this review that evaluated changes in both medication and counseling delivery did not evaluate quit rates [53]. This may result from poor compatibility between information routinely documented in the medical record and information needed to draw conclusions about an implementation strategy’s clinical effectiveness, such as changes in tobacco smoking. It could be challenging to embed data collection for research purposes within a clinical environment, where research data may not align with clinical data entered by providers and where patients may not be accustomed to completing additional assessments for research. Strategic investments are needed to develop infrastructure in real-world health-care settings that can measure changes in smoking behavior. The Learning Health Systems model [83, 84] can support efforts to integrate data collection with practice to inform both clinical care (e.g., by tracking smoking changes over time) and system-level efforts to improve the public health impact of tobacco interventions.

Finally, enhancing the quality of implementation trial design, analyses, and reporting would support exploration of potential moderators and mediators of tobacco intervention changes in mental health settings. In regards to trial design, sufficiently-powered RCTs are the gold standard for evaluating which implementation strategies are superior to others for producing a certain outcome but are not always feasible in clinical settings [85]. Studies that are not testing formal hypotheses can still contribute to testing and developing theories about why and how implementation strategies work by reporting on implementation processes and outcomes, such as how barriers were identified, why strategies were selected, how strategies were tailored to local settings, and whether strategies led to clinical practice change [85]. It is recommended that implementation efforts, starting with identifying unmet needs through evaluating outcomes, be conducted iteratively to allow persistent areas of unmet need to be identified and addressed [86]. Building iterative evaluations of implementation processes into protocols for RCTs could enhance researchers’ abilities to draw conclusions about which implementation strategies produce the greatest tobacco intervention improvements in mental health settings, as well as why and how they promote such improvements.

Using analyses that can detect moderating and mediating variables are also critical to enhance our understanding of how setting type and behavior change mechanisms influence implementation strategy impact on tobacco intervention delivery in mental health settings. In a systematic review, Williams [87] identified nine implementation-focused RCTs that evaluated mediators of change at the system (organizational climate, barrier type, resources, support from leadership or opinion leaders) and individual (attitudes, self-efficacy, readiness to change) level in mental health settings and did not identify any significant mediators. Williams recommended statistical analyses like multilevel mediational analyses, which can also permit identification of moderators, as one technique to expand detection of significant mediators [87]. For social scientists, Andrew F. Hayes’ book and website (https://www.afhayes.com/index.html) provide an excellent and practical introduction to these analyses [88]. Williams also encourages researchers to test theories about which mediators may produce desired changes and why mediators may or may not produce such changes [87]. Five studies [52, 53, 59, 63, 64] included in this review provided a theoretical justification for their implementation approach and two [59, 64] formally evaluated proposed mechanisms of change. Both studies used an organizational change framework but one evaluated leadership perceptions of organizational culture [59] and the other evaluated provider perceptions of organizational culture and barriers to change [64]. Conclusions about how implementation strategies change tobacco intervention practices in mental health settings can only be drawn if there is a valid assessment of the theorized construct; in other words, the level (individual, organizational, policy) of theory needs to align with the level of measurement [87]. A social cognition theory, like the theory of planned behavior [79], or implementation framework like CFIR [34], would have provided a better theoretical justification for assessments of provider views. Qualitative methods used alone or with quantitative methods can also provide rich information about the impact of the implementation strategies and relationship between actual and theorized mediators [89].

Finally, Proctor and colleagues [31] recommend that implementation researchers identify and report the implementation actors, strategies, targets, phase, dose, outcomes, and theoretical justification. Most studies included in this review identified actors, strategies, and outcomes. In regard to targets, one evaluated organizational factors as possible moderators of implementation processes [59] and another evaluated whether tobacco cessation medication use and counseling sessions completed by patients mediated abstinence [57]. Implementation dose was not evaluated in studies included in this review, and implementation literature on the dose-response effect is mixed: the amount of time spent in implementation activities was associated with improved delivery of medications for alcohol use disorder in substance use and primary care clinics [81], but was not associated with therapist delivery of cognitive behavioral therapy in primary care clinics [90]. Documenting implementation dose in future implementation trials could expand the field’s understanding of how dose impacts practice change. Worksheets [36] and free applications such as toggl© (https://toggl.com/) can easily facilitate time tracking. Reporting all seven of Proctor’s elements in future implementation trials will help advance theories and inform hypotheses about what and how implementation strategies work for improving delivery of tobacco interventions in mental health settings [31].

## Conclusion

Effects of implementation strategies on tobacco interventions may be moderated by mental health setting and mediated by behavior change mechanisms that align with the treatment philosophy and tobacco intervention barriers unique to specific settings (inpatient vs. outpatient mental health). Based on findings from this review, it is challenging to recommend a set of strategies for improving tobacco interventions in all types of mental health settings. Further, it is impossible to say for sure whether these proposed moderators and mediators were at play given the absence of quantitative analyses designed to test for these effects and qualitative data from providers about the perceived effectiveness of specific strategies in local settings. Based on data synthesized in this review, strategies that *engage consumers* in outpatient settings and *change infrastructure* in inpatient settings may be optimal for improving reach of tobacco cessation medications, in particular. Using strategies within the domain *adapt and tailor to context* would also be useful to include. Even though just one study [64] included in this review described tailoring implementation strategies to local needs, a prior systematic review of practice facilitation trials found that effect sizes for health care practice improvements were greater when strategies were tailored to local settings [49]. In sum, future implementation trials that incorporate recommendations from this review will be well-suited to explain the complex implementation processes that improve tobacco intervention in different types of mental health settings.

## Supplementary Information

Supplementary Material 1.

Supplementary Material 2.

Supplementary Material 3.

Supplementary Material 4.

## Data Availability

The datasets used and/or analyzed during the current study are available from the corresponding author on reasonable request.

## Abbreviations

4As: Advise, Assess, Assist, Arrange
5As: Ask, Advise, Assess, Assist, Arrange
ATTOC: Addressing tobacco through organizational change
BCW: Behavior change wheel
CFIR: Consolidated framework for implementation research
CI: Confidence interval
COM-B: Capability, opportunity, motivation model of behavior change
ERIC: Expert recommendations for implementing change
FDA: Food and drug administration
JCAHO: Joint commission on accreditation of healthcare organizations
NRT: Nicotine replacement therapy
OR: Odds ratio
PRISMA: Preferred reporting items for systematic reviews and meta-analyses
RCT: Randomized controlled trials
RE-AIM: Reach, effectiveness, adoption, implementation, and maintenance
RoB-2: Risk of bias
RoBINS-I: Risk Of bias in non-randomized studies of interventions
SWiM: Synthesis without meta-analysis
TTTF: Taking texas tobacco free
US: United States

## Footnotes

1 We updated the review protocol in response to feedback from an implementation science expert to use the behavior change wheel instead of the behavior change taxonomy because the former is better suited for identifying mechanisms of change for strategies that focus on providers or systems.

2 Though not part of the original registered review protocol, we also categorized implementation strategies according to the ERIC taxonomy in response to feedback from a colleague who reviewed an earlier version of this manuscript and to align with Proctor and colleagues’ [31] implementation strategy reporting recommendations.

3 Traditionally, Adoption is a measure of provider uptake of the evidence-based intervention, like the changes in the proportion of providers prescribing quit medication at least once over time, promoted by the implementation strategy [35, 36]. We use a broader definition provided by Holtrop [37], which emphasizes how adoption can be measured on an individual, clinic, or policy level and capture additional outcomes, like adoption of a policy to reduce smoking. We also deferred to authors’ language when deciding whether to categorize a study as having measured adoption to provide as comprehensive an overview of relevant literature as possible.

4 Nitturi^47^ asked about *Assisting* in a general way, “With regard to patients that you saw last month who smoked, did you assist them to quit by providing treatment or making a referral for treatment?”
