# Supplementary material for: Implementation strategies to increase tobacco treatment in mental health settings: a systematic review
Source: BMC Psychiatry. 2025 Oct 8;25:945. doi: 10.1186/s12888-025-07248-7 (PMC12506521; doi:10.1186/s12888-025-07248-7)
Supplement: Supplementary file 1 — Supplementary Material 1. [file 12888_2025_7248_MOESM1_ESM.docx]

|  |  |  |  |  |  |  |  |
| --- | --- | --- | --- | --- | --- | --- | --- |
| **Article** | **Bias due to confounding** | **Support for judgement** | **Bias in selection of participants into the study** | **Support for judgement** | **Bias in classification of interventions** | **Support for judgement** | **Bias due to deviations from intended interventions** |
| **Carrillo et al., 2016** | Moderate | Confounding expected, but appropriately measured and controlled for. Reliability and validity of measurement of important domains were sufficient. Cochran-Mantel-Haenszel test evaluated outcome changes over time and controlled for psychiatric diagnosis. | Low | All eligible participants were included and all eligible participants had the same follow-up and implementation strategy start date. | Low | Exposure to implementation strategy was well defined and based exclusively on information collected at time of strategy. Center for Medicare and Medicaid Services (CMS) rule was implemented on a single day. | Low |
| **Chen et al., 2018** | Serious | Confounds, including time, were not evaluated or controlled for. | Moderate | Psychiatric diagnosis may have influenced which clinicians had access to decision support and, thus, how clinicians provided treatment; all eligible participants had the same follow-up and implementation strategy start date, though it is unclear what this date was for all activities. | Serious | Implementation period for different activities, as well as pre and post-implementation periods, are not well-defined: One implementation activity occurred for approximately 30 days during two years. It is unclear when the other activity was implemented. | No information |
| **Correa-Fernández et al., 2019** | Serious | “Multilevel” models were used but levels were not specified; baseline organizational characteristics and employee access to training were “individually” controlled for. | Low | All eligible participants were given the opportunity to participate; start of follow-up and start of implementation strategy coincided for each participant. | Low | Implementation strategy status well-defined and based on information collected at strategy initiation. | Low |
| **Hollen et al., 2010** | Serious | At least one important domain was not appropriately controlled for: Hospitals that implemented smoking bans had fewer beds, but this was not accounted for statistically. | Low | No indication of selection bias; follow-up consistent across participants. | Low | Implementation status well-defined and determined *a priori*. | Low |
| **Huddlestone et al., 2018** | Serious | Some confounds were not measured, like psychiatric diagnosis; secular trends were not explored. | Low | All eligible participants were included, and the start of the implementation strategy and follow-up coincided for all participants. | Low | Implementation status is well-defined and based on information collected at time of implementation. | Moderate |
| **Kanter Bax et al., 2020** | Serious | Secular trends and other potential confounds not were not measured or evaluated. | Low | All eligible participants appeared to be included and had the same follow-up period. | Serious | The start, end, and nature of each PSDA occurred is not clear. | Moderate |
| **Lappin et al., 2020** | Serious | The impact of time was not evaluated. | Low | All eligible patients were included; start of follow-up coincided for providers and patients. | Low | Intervention status is well defined and based on information collected at time of intervention. | No information |
| **Muladore et al., 2018** | Serious | Secular trends not reported. | Moderate | Selection into study based on tobacco use may have been related to implementation strategy and outcome. Unclear how past 30-day tobacco use was assessed to determine inclusion. Per authors, “Data about tobacco use were based on what was available for each patient. Of note, many of the tobacco use histories completed by nurses at admission were incomplete to varying degrees.” | Low | Implementation period well-defined. | Low |
| **Nitturi et al., 2021** | Serious | Secular trend not evaluated. Other potential confounds, like length of involvement in train-the-trainer program and organizational characteristics, not evaluated.  “Program implementation spanned the fall of 2020 through the spring of 2021 with start and end dates varying by participating LMHA based on their preferences and progress [range = 4-6 months]. | Low | All eligible sites were given the opportunity to participate; start of follow-up and implementation activity coincided. All LMHAs were included in earlier TTF efforts. | Low | Implementation status was well-defined and based on information collected at initiation. | Low |
| **Nitturi et al., 2021a** | Moderate | Confounding expected but appropriately measured and controlled for with valid measures. Clustered by LMHA; models adjusted for baseline covariates (baseline LMHA size measures, ORIC score, or both, depending on outcome of model) that appeared to be validly measured. Moderators examined; time taken into account with matched variables. | Moderate | Selection into study was determined based on readiness to change and enrollment capacity and, thus, may have been related to receipt of implementation strategy and outcome. | Low | Implementation status is well-defined and appears to be based on information collected as baseline. | Low |
| **Okoli et al., 2018** | Moderate | Secular trends evaluated, but other confounds (like psychiatric diagnosis) not included in analyses. | Low | All eligible participants included; start of follow-up and start of strategy aligned for all participants. | Low | Implementation status well-defined and based solely on information collected at time of intervention. | Low |
| **Parker et al., 2012** | Serious | Confounds like diagnosis, number of counseling sessions, and treatment setting not accounted for in data analysis. | Serious | Start of follow-up and implementation activity did not align for all participating sites, so some patients had longer exposure to implementation activities than others. “Seven months into the study, two more acute wards (44 beds) were incorporated because the ward managers expressed an interest in participating (total of beds = 129).” | Serious | 9-month project period, but it is unclear when exactly baseline data was collected, when the service was established, when follow-up data was collected, and what time periods were covered during baseline and follow-up data collection. | Low |
| **Scharf et al., 2011** | Moderate | Secular trends evaluated and other confounds (e.g., hospital unit) evaluated and measured reliably. | Low | All eligible participants included and start of follow-up and start of implementation activity coincided for all participants | Low | Implementation status well-defined. Ban implemented on one day and training occurred for one month prior to the ban and 4 months after. | Low |
| **Scheeres et al., 2020** | Serious | Repeated cross-sectional design. At least one important domain was not appropriately controlled for (facility) and differences in pre- and post-implementation groups were not explored. Secular trends not evaluated. | Low | All eligible participants included, and the start of follow-up and implementation strategy coincided for all participants. | Low | Implementation status well-defined and based on information collected at time of intervention. | Low |
| **Wye et al., 2017** | Moderate | Repeated cross-sectional; confounds, including time, reliably assessed, evaluated, and controlled for in analyses. | Low | All eligible participants included and start of follow-up and implementation strategy coincided for all participants. | Low | Implementation strategy well-defined and based solely on information collected at time of implementation. | Low |

|  |  |  |  |  |  |  |  |  |
| --- | --- | --- | --- | --- | --- | --- | --- | --- |
| **Article** | **Support for judgement** | **Bias due to missing data** | **Support for judgement** | **Bias in measurement of outcomes** | **Support for judgement** | **Bias in selection of the reported result** | **Support for judgement** | **Overall risk of bias** |
| **Carillo et al., 2016** | None reported. Any deviations would reflect usual practice. | Serious | More missing data in pre-implementation group for all outcomes, which could be due to lack of documentation requirements addressed by strategy, but other predictors of missingness not explored; nature of missing data means that risk of bias cannot be removed through appropriate analysis. | Low | Outcomes obtained from medical records and comparable before and after implementation strategy; outcomes unlikely to be influenced by knowledge of implementation strategy; error in outcome measurement is not expected to be related to implementation strategy. | Moderate | No pre-registered statistical plan; outcomes are clearly defined; no indication of selective outcome reporting. | Serious |
| **Chen et al., 2018** |  | Low | Data obtained from medical record and reasonably complete. Less than 1% of data was missing. | Low | Outcome assessments comparable over time and outcome measurement error unlikely to be influenced by knowledge of implementation strategy status. | Moderate | Outcome measurements and analyses are consistent with an a priori plan, but this plan does not specify pre and post-implementation periods; no indication of selective outcome reporting from multiple analyses or subgroups. | Serious |
| **CorreaFernandez et al., 2019** | Deviations not reported; possible deviations were likely related to characteristics of real-world practice. | Moderate | Pre-and post implementation survey samples were different, but systematic differences were not explored, so missing data risk of bias cannot be ruled out. | Serious | Outcome measures were subjective and may have been influenced by awareness of implementation efforts. | Serious | There is a high risk of selective reporting: multilevel models individually adjusted for covariates, but adjusted odds ratios not reported: “The patterns of the results remained largely unchanged after individually controlling for organizational and program-related characteristics (data not shown; available from corresponding author upon request).” CK reached out to primary author for data and has yet to hear back. | Serious |
| **Hollen et al., 2010** | Deviations not reported; any deviations likely due to usual practice and unlikely to impact outcomes. | Low | Data were reasonably complete and missingness was similar between both groups. 3 of 70 hospitals were missing data on one item that is not expected to be related to outcome. | Low | Outcome assessment methods were comparable across groups and limited subjectivity of self-reported smoking restrictions; any measurement error is unlikely to be influenced by research question. | Low | Reported data analysis plan; all reported results correspond to intended outcomes, analyses, and sub cohorts. | Serious |
| **Huddlestone et al., 2018** | There were deviations from the implementation strategy beyond that of usual practice that likely impacted the treatment provision outcome. | Low | Data were reasonably complete. | Low | Methods of outcome assessment were comparable pre- and post-implementation; one outcome measure (self-reported NRT delivery) could have been influenced by knowledge of implementation strategy, but medical record NRT data was also obtained and reported and revealed incongruence; measurement outcome error unlikely to be related to implementation strategy. | Serious | Outcome measurement and analysis appear consistent with *a priori* plan; there is a high risk of selective reporting because statistical tests and significance tests are not reported for any outcome; number of cigarettes smoked after admission was a planned outcome but not collected. | Serious |
| **Kanter Bax et al., 2020** | The referral process in the electronic health record was planned, but not included in PDSA cycle descriptions; its implementation was also unexpectedly delayed, which likely impacted the main outcome measure (referrals to smoking treatment). | No information |  | Critical | Outcome assessment appears to be comparable across evaluation cycles but was subjective and not clearly described “For measurement, smoking cessation referrals were counted by feedback through various sources: Trusts' performance data, confirmation from smoking cessation providers of referrals received, and immediate feedback from care coordinators.” | Serious | No statistical analyses described or reported; there is a risk of selective reporting, “those who relapsed into smoking after successful cessation or transitioned from the service were removed from the outcome [smoking cessation referral] numbers.” | Critical |
| **Lappin et al., 2020** | No indication of deviation occurring. | Low | Data were reasonably complete. | Low | Data obtained retrospectively from medical record. Outcome assessments comparable over time and outcome measurement error unlikely to be influenced by knowledge of implementation strategy status. | Low | Unclear which outcomes were pre-planned, and which were exploratory; no indication of selection of reported results from multiple analyses or subgroups. | Serious |
| **Muladore et al., 2018** | No deviations reported; any that occurred were likely related to usual practice and unlikely to impact outcome. | No information |  | Moderate | Outcomes were assessed the same way pre- and post-implementation but may have been influenced by provider awareness of implementation; any error is not likely to be related to implementation status. | Moderate | Unclear which outcomes were pre-planned, and which were exploratory; no indication of selection of reported results from multiple analyses or subgroups. | Serious |
| **Nitturi et al., 2021** | No reported deviations; deviations likely to be related to usual practice. | Low | Data was available for all participants. | Moderate | Outcome assessment was comparable over time, but was self-reported and may have been influenced by knowledge of implementation strategy. | Serious | Unclear how adoption outcome (35.02%) was obtained and does not match data plan formula.  Small sample size (n=10), so no statistical tests reported. | Serious |
| **Nitturi et al., 2021a** | No indication of deviation, but deviations would likely be related to usual practice. | Moderate | 10% had missing follow-up data and were not included in analysis; no evaluation of how these sites differed from sites with complete data. | Serious | Outcome was subjective (clinician-report) and may have been influenced by knowledge of implementation strategy. | Moderate | Outcome measures and analyses appear consistent with an a priori plan; no indication of selection of reported results from multiple analyses or subgroups. | Serious |
| **Okoli et al., 2018** | No deviations reported; if occurred, would likely be related to usual practice. | Moderate | Data were reasonably complete (6% missing for Ask outcome); missing rate for other outcomes not reported; predictors of missing data were not evaluated. | Low | Data obtained retrospectively from medical record by a hospital coder; unlikely to have been influenced by implementations status. | Low | *A priori* plan; no indication of selective reporting. | Moderate |
| **Parker et al., 2012** | Deviations not reported, but likely to be related to usual practice. | Low | Data was reasonably complete. | Low | NRT data obtained from medical record and abstinence confirmed by carbon monoxide readings. Unclear how smoking reduction was reported. | Serious | Main outcomes not pre-specified; no a priori data analysis plan. “The aim of this pragmatic pilot project was to develop and implement a tailored tobacco dependence service in mental health settings and to assess its impact, as well as barriers and facilitators to implementation.” | Serious |
| **Scharf et al., 2011** | No deviations reported; deviations likely to reflect usual practice and not impact outcome. | Low | Data reasonably complete. | Low | Retrospective chart review, so unlikely to have been influenced by knowledge of implementation status; any outcome measurement error not likely due to implementation status; outcome assessment method comparable over time and across units. | Moderate | No a priori data analysis plan, but all hypotheses addressed; no indication of selection of reported result from multiple analyses or subgroups. | Moderate |
| **Scheeres et al., 2020** | No indication of deviation; deviations likely to reflect usual practice and unlikely to influence outcome. | Low | Data were reasonably complete. | Low | Retrospective administrative data review; outcome assessment was comparable over time and unlikely to have been influenced by knowledge of implementation status; and any outcome measurement error is unlikely to be related to implementation status. | Low | No a priori data analysis plan; no indication of selection of reported results from multiple analyses or by subgroup | Serious |
| **Wye et al., 2017** | No deviations reported; any deviations likely related to usual practice and unlikely to impact outcome. | Low | Data was reasonably complete. | Low | Retrospective chart review; outcome assessment comparable over time and unlikely to be influenced by knowledge of implementation status; any outcome error unlikely to be related to implementation status. | Moderate | No a priori data analysis plan, but analyses are clearly described; no indication of selection of results from multiple analyses or subgroups. | Moderate |
