# Supplementary material for: Implementation strategies to increase tobacco treatment in mental health settings: a systematic review
Source: BMC Psychiatry. 2025 Oct 8;25:945. doi: 10.1186/s12888-025-07248-7 (PMC12506521; doi:10.1186/s12888-025-07248-7)
Supplement: Supplementary file 2 — Supplementary Material 2. [file 12888_2025_7248_MOESM2_ESM.docx]

| **Article** | **Randomizati-on Process** | **Support for judgement** | **Timing of recruitment** | **Support for judgement** | **Bias due to deviations from intended interventions** | **Support for judgement** | **Bias due to missing outcome data** | **Support for judgement** | **Bias in measurement of outcomes** | **Support for judgement** | **Bias in selection of the reported result** | **Support for judgement** | **Overall risk of bias** |  |  |
| --- | --- | --- | --- | --- | --- | --- | --- | --- | --- | --- | --- | --- | --- | --- | --- |
| **Brunette et al., 2015** | Some concerns | No details about randomization procedure. Baseline differences in age and mental health diagnoses such that patients with providers who participated in video were slightly younger and had higher prevalence of schizophrenia, anxiety, and personality disorders. | N/A | N/A | Low | No reported deviations; any deviations were likely related to usual practice and unrelated to outcome. | Low | No indication of missing data. | Low | Retrospective chart review: low likelihood that intervention assignment influenced outcome assessment. | Some concerns | No indication of a priori data analysis plan. | Some concerns |  |  |
| **McFall et al., 2005** | Some concerns | No details about randomization procedure. | N/A | N/A | Low | No blinding | Low | Data were reasonably complete. | Low |  | Some concerns | No indication of a priori data analysis plan. | Some concerns |  |  |
| **McFall et al., 2010** | Some concerns | Used adaptive randomization procedure and telephone randomization system. No details provided about concealment of allocation sequence. | N/A | N/A | Some concerns | Patients and providers not blinded. No information about deviations from intervention, but any deviations were likely to be attributed to usual care and unlikely to have systematically impacted outcome. | Low | "10% missing data; dropouts more likely to be younger, have higher PTSD symptom scores, served in Iraq or Afghanistan, and have a history of alcohol abuse or dependence. Because dropout number was similar between intervention groups, outcome data was dichotomous, and outcomes reported in odds ratios, this is an example of a special case of low risk of bias due to missingness (Higgins et al., 2019, p. 40)." | Low |  | Low |  | Some concerns |  |  |
| **Dixon et al., 2009** | Some concerns | No details about randomization procedure. | N/A | N/A | Some concerns | No information about deviation from intended intervention; deviations likely related to usual practice and unlikely to impact outcome. Unclear if providers or patients were blinded to intervention status. | Some concerns | Retained 77% of data at 12-month follow-up. No indication of whether missingness was predicted by other variables. | Low | Unclear if patients were blinded to intervention received, but seems likely they were unaware since the intervention focused on provider behavior. | Some concerns | No indication of a priori data analysis plan. | Some concerns |  |  |
| **Schnoll et al., 2023** | Some concerns | Unknown whether the allocation sequence was concealed. Baseline differences between clusters not assessed. | Some concerns | Unclear whether particpants were recruited before or after cluster randomization. Several differences in baseline characteristics of patients and providers between groups. | Low | No blinding; appropriate analysis was used to estimate the effect of group assignment. | Low | Data reasonably complete. | Low | Data obtained from electronic health record. Knowledge of group is unlikely to have influenced outcomes. | Low | Data analysis consistent with trial registry data analysis plan. | Some concerns |  |  |
