# Supplementary material for: Implementation strategies to increase tobacco treatment in mental health settings: a systematic review
Source: BMC Psychiatry. 2025 Oct 8;25:945. doi: 10.1186/s12888-025-07248-7 (PMC12506521; doi:10.1186/s12888-025-07248-7)
Supplement: Supplementary file 3 — Supplementary Material 3. [file 12888_2025_7248_MOESM3_ESM.docx]

| **Authors** | **Design** | **Population^a^** | **Quit EBPs** | **Implementation length** | **ERIC implementation activities^b^** | **RE-AIM outcomes** |
| --- | --- | --- | --- | --- | --- | --- |
| Brunette et al., 2015 (USA) | Parallel RCT | 14,615 psychiatric outpatients with schizophrenia, bipolar, depression, anxiety, substance use, personality, and other disorders | *Assist* with quit medication (NRT and varenicline) | 50 minutes | **Use evaluative and iterative strategies**  Audit and provide feedback  **Train and educate stakeholders**  Develop educational materials  Distribute educational materials  Conduct educational outreach visits | Reach: At 9-month follow-up, there was an increase in *Assisting* with NRT prescriptions, per Medicaid claims data for all patients with a serious mental illness, (coefficient = 0.53, *p <* .01) after strategy. NRT increased more after virtual compared to in-person modality (coefficient = -0.62, *p* = .02) such that there was a surge, followed by a decrease in NRT prescriptions after in-person, and an increase in NRT after virtual. After strategy, varenicline prescriptions declined at a slower rate than pre-strategy, indicating a small relative increase (trend change coefficient = 0.09, p = .04). Type of modality did not impact varenicline prescribing trends. |
| Carrillo et al., 2017^§^  (USA) | Repeated cross-sectional | 630 psychiatric inpatients with depressive disorders, schizophrenia spectrum and other psychotic disorders, bipolar and related disorders, substance-related and addictive disorders, or trauma-and-stressor-related-disorders | *Ask* about tobacco use, *Assist* with referrals, quit counseling, and quit medication (NRT and bupropion) | Tobacco use assessment effective January 1, 2015 | **Use evaluative and iterative strategies**  Develop and implement tools for quality monitoring  **Train and educate stakeholders**  Work with educational institutions  **Support clinicians**  Facilitate relay of clinical data to providers  **Utilize financial strategies**  Develop disincentives  **Change infrastructure**  Mandate change  Change record systems | Reach: At 1-year follow-up, 12% absolute increase in *Asking* about tobacco use (85% to 97%; OR = 5.19, p < .001) per medical record for psychiatric inpatients during study period. For inpatients identified as smokers, there was a 70% absolute increase in *Assisting* with referrals to quit counseling (4% to 74%, OR = 7.26, p < .001) and 59% absolute increase in receipt of quit counseling (8% to 67; OR = 22.99, p < .001). 36% absolute increase in referral for NRT or NRT+bupropion (32% to 68%, OR = 6.29, p < .001), but no statistically significant change in actual receipt of quit medication (NRT or bupropion) (24% to 35%, OR = 1.59, p = .08). |
| Chen et al., 2018^§^ (USA) | Repeated cross-sectional | 6,467 psychiatric outpatients with schizophrenia, schizoaffective, mood, posttraumatic stress, or borderline personality disorder | *Assist* with quit medication (NRT and varenicline) | 2 months of decision support + annual 1-hour training | **Use evaluative and iterative strategies**  Audit and provide feedback  **Train and educate stakeholders**  Develop educational materials  Distribute educational materials  Conduct educational outreach visits  **Support clinicians**  Facilitate relay of clinical data to providers  **Engage consumers**  Prepare patients/consumers to be active participants | Reach: At 2-year follow-up, 13% absolute increase in proportion of smoking patients prescribed NRT or varenicline per pharmacy database (5% to 18%, z = 12.3, *p* ≤ .001)  Effectiveness: At 2-year follow-up, 3% absolute decrease in smoking prevalence (57% to 54%, z= 2.63, *p* ≤ .001). |
| Correa-Fernández 2019^c^ (USA) | Repeated cross-sectional | 1,079 psychiatric providers | *Ask, Advise* quitting*, Assess* interest in quitting*, Assist* with quit counseling and quit medication (NRT vs. bupropion or varenicline), *Arrange* follow-up | 36 months | **Use evaluative and iterative strategies**  Assess for readiness and identify barriers  Audit and provide feedback  **Provide interactive assistance**  Facilitation  Provide local technical assistance  **Develop stakeholder interrelationships**  Identify and prepare champions  Recruit, designate and train leadership  Obtain formal commitments  Capture and share local knowledge  **Train and educate stakeholders**  Provide ongoing consultation  Develop educational materials  Make training dynamic  Distribute educational materials  Conduct educational meetings  Conduct educational outreach visits  **Engage consumers**  Prepare patients/consumers to be active participants  Use mass media  **Change infrastructure**  Mandate change  Change physical structure and equipment | Adoption: At 6-month follow-up, 13% absolute increase in proportion of providers who *Asked* all patients about cigarette use (48% to 61%, OR = 1.75, *p* < .001). Absolute increases were as follows for proportion of providers who engaged in the following in past month for patients who smoked: 16% for *Advising* (58% to 74%, OR = 2.04, *p* < .001), 18% for *Assessing* (56% to 74% OR = 2.35, *p* < .001), 31% for *Assisting* (with counseling or medication; 31% to 61%, OR = 3.75, p < .001): no change in *Assist* with quit counseling (OR = 1.15, *p* = .28, *p* = .28), increase in *Assist* with NRT (no percentages reported; OR = 3.29, *p* < .001), and increase in non-NRT smoking cessation medication (no percentages reported; OR = 1.52, *p* = .04). 19% increase in *Arranging* (26% to 45%, OR = 2.38, *p* < .001). Confidence intervals not reported.  Absolute increase (range 35-53%) in provider-reported past year receipt of tobacco education (OR range = 3.36 – 18.89, ps < .0001). Confidence intervals not reported. |
| Dixon et al., 2009^§^ (USA) | RCT | Unknown number of psychiatrists  304 psychiatric outpatients with schizophrenia spectrum disorders, affective psychoses, or other psychoses | *Ask, Advise* quitting, *Assess* interest in quitting, *Assist* with quit medication (NRT vs. wellbutrin or chantix) or group counseling, *Arrange* follow-up | 6 months | **Develop stakeholder interrelationships**  Conduct local consensus discussions  Develop academic partnerships  **Train and educate stakeholders**  Provide ongoing consultation  Develop educational materials  Distribute educational materials  Conduct educational outreach visits  **Support clinicians**  Remind clinicians  **Engage consumers**  Prepare patients/consumers to be active participants  **Change infrastructure**  Change record systems | Reach: At 12 month follow-up, increases in proportion of smoking patients reporting 4As during most recent psychiatry visit: *Assist*  with NRT (8% to 24%; 16% absolute increase, X^2^(1) = 15.75 *p* <.001), Wellbutrin or Chantix (4% to 13%; 9% absolute increase X^2^(1) = 10.03, *p* = .002), and with group therapy referral (6% to 17%, 11% absolute increase, X^2^(1) = 8.71, *p* = .003).  Effectiveness: At 12-month follow-up, 5% decrease in proportion of patients reporting past week smoking (99% to 94%, *p* = .02) and 10% reduction in average weekly cigarettes smoked per patient (138 to 125*, p* = .05). At 6-month follow-up, smoking changes did not differ by condition (ps > .36; 12-month time by condition interaction not reported). |
| Hollen 2010 (USA) | Observational cohort study | 70 psychiatric hospital administrators | *Assist* with quit medication (NRT or bupropion) | Date of smoking ban varied across sites | **Train and educate stakeholders**  Conduct educational outreach visits  **Change infrastructure**  Mandate change | Adoption: Among hospitals that went smoke-free between 2006 and 2008, there was a 32% absolute increase in proportion of hospitals that offered “pharmacotherapy” (included bupropion but unsure what other medications) (50 to 82%, *p* < .05). Also non-significant increases in offerings of different types of NRT (increases ranged from 21% for lozenges to 3% for gum).  For hospitals that still allowed smoking, there was not a significant change in NRT or other pharmacotherapy offered.  Among hospitals that went smoke-free between 2006 and 2008, there was no change in number of smoking-related staff training; there was a significant decrease in number of trainings on smoking assessment (29% decrease, 67% to 38%) and smoking treatment (31% decrease, 69% to 38%) offered at hospitals that kept smoking (*ps* < .01). |
| Huddlestone 2018^§^ (UK) | Repeated cross-sectional | 315 psychiatric inpatients with primarily schizophrenia, bipolar, and psychosis | *Ask* about smoking, *Advise* quitting, *Assist* with quit medication (NRT) or referral | Smoking ban and mandated smoking assessment effective April 2016 | **Use evaluative and iterative strategies**  Obtain and use patients/consumers and family feedback  **Support Clinicians**  Create new clinical teams  **Change infrastructure**  Mandate change | Reach: At 5-month follow-up, 10% absolute decrease in proportion of patients with provider medical record documentation of being *Asked* about smoking (94% to 84%). Proportion of smoking patients with documentation of *Advice* decreased by 9% (81% to 72%), referral to smoking cessation services increased 30% after an in-house referral option was available (23% to 60%), and staff documentation of NRT provision in health record increased 51% (7% to 58%). Pharmacy-confirmed NRT prescriptions increased by 10% (7% to 17%). No statistical tests reported. |
| Kanter-Bax 2020 (UK) | Repeated cross-sectional for reach outcome  Cohort for effectiveness | Unknown *n* outpatients with first episode psychosis | *Ask* about smoking, *Assess* interest in quitting, *Assist* with referrals | 6 months | **Use evaluative and iterative strategies**  Audit and provide feedback  Develop a formal implementation blueprint  Obtain and use patients/consumers and family feedback  Conduct cyclical small tests of change  **Develop stakeholder interrelationships**  Organize clinician implementation team meetings  Use an implementation advisor  **Train and educate stakeholders**  Make training dynamic  Distribute educational materials  Conduct educational outreach visits  **Support Clinicians**  Remind clinicians  Create new clinical teams  **Engage consumers**  Involve patients/consumers and family members  Prepare patients/consumers to be active participants  **Change infrastructure**  Change record systems  Change service sites | Reach: At 24-month follow-up, increase in proportion of total patient sample *Asked* about smoking and *Assessed* for treatment (both were part of one questionnaire) to quit from <1% to 77%. Percentage of completed referrals for smoking patients to national tobacco treatment increased from 1% to 18%. No statistical tests reported.  Effectiveness: 26% of baseline patients who smoked had quit by 24-month follow-up. |
| Lappin 2020 (Australia) | Repeated cross-sectional | 423 psychiatric inpatients with primarily psychotic spectrum disorders | *Ask* about smoking, *Assist* with quit medication (NRT) | 3 months | **Develop stakeholder interrelationships**  Identify and prepare champions  **Train and educate stakeholders**  Conduct ongoing training  **Support clinicians**  Revise professional roles  **Utilize financial strategies**  Alter incentive/allowance structures  **Change infrastructure**  Mandate change  Change physical structure and equipment | Reach: After 3 months, per medical record for inpatient sample during study period, there was a 9% absolute increase in patients with documentation of being *Asked* about smoking (2% to 11%, *p* ≤ .001). 16% absolute increase in proportion of all inpatients prescribed NRT (*Assist*) (11% to 27%, *p* ≤ .001). |
| McFall 2005 (USA) | RCT | Integrated care = 33 PTSD outpatients  Usual care (PTSD treatment + referral to smoking cessation clinic)= 33 PTSD outpatients | *Assist* with quit counseling | Unknown implementation length for integrated care | **Train and educate stakeholders**  Provide ongoing consultation  Distribute education materials  Conduct educational outreach visits  **Support clinicians**  Revise professional roles | Implementation: Smoking cessation behavioral counseling sessions were greater in integrated (mean = 5, SD = 1.2) vs. usual care (mean = 3, SD = 2.1, z = 5.35, *p* < .0001).  Effectiveness: No significant difference in 7-day point prevalence abstinence (12% integrated care vs. 3% usual care, (χ2=1.66, df=1, *p*=0.20, two-tailed). At 2,4, 6 and, 9-month follow-ups, greater odds of being quit in integrated care vs. usual care (OR = 5.23 [1.76-15.54], *p* < .02). |
| McFall 2010^d^ (USA) | RCT | Integrated care = 472 PTSD outpatients  Usual care (PTSD treatment + referral to smoking cessation clinic)= = 471 PTSD outpatients | *Assist* with quit counseling | 36 months | **Train and educate stakeholders**  Distribute educational materials  Use train-the-trainer strategies  Conduct educational outreach visits  **Support clinicians**  Remind clinicians  Revise professional roles | Implementation: Median number of smoking cessation behavioral counseling sessions were greater in IC than usual care (8 vs. 1 session, *p* < .001).  Effectiveness: IC had higher bioverified 12-month prolonged abstinence than usual care (9% vs. 5%, AOR = 2.26, 95% CI, 1.30-3.91; *p* =.004). 18-month bioverified 30-day point prevalence abstinence was greater for IC than usual care (17% vs. 9%, p < .001, OR = 2.17, 95% CI, 1.56-3.03; *p* < .001). |
| Muladore 2018^§^ (USA) | Repeated cross-sectional | 54 psychiatric inpatients with depression, anxiety, and substance use | *Assist* with quit counseling and quit medication (NRT gum) | 1 day | **Train and educate stakeholders**  Make training dynamic  Distribute educational materials  Conduct educational outreach visits  **Support clinicians**  Facilitate relay of clinical data to providers  **Change infrastructure**  Mandate change | Reach: At 31-day follow-up, no statistically significant absolute increase in proportion of smoking inpatients (seen by consenting providers) with documentation of being *Assisted* with both NRT gum + counseling (9%, 14% to 23%, *p* = .06) or only NRT gum (20%, 50% to 70%, *p* = .13). |
| Nitturi 2021 (USA) | Cohort | 10 non-prescribing providers | None | 6-9 months, depending on site | **Use evaluative and iterative strategies**  Audit and provide feedback  Obtain and use patients/consumers and family feedback  **Develop stakeholder interrelationships**  Identify and prepare champions  Obtain formal commitments  Model and stimulate change  Develop academic partnerships  **Train and educate stakeholders**  Distribute educational materials | Adoption: At 6-month follow-up, 35% absolute increase in number of tobacco education sessions delivered by champions to employees. |
| Nitturi 2021a (USA) | Repeated cross-sectional | 1,237 providers | *Ask, Advise* quitting, *Assess* interest in quitting*, Assist* with quit counseling, referral, or quit medication, *Arrange* follow-up | 6 months | See Correa-Fernández 2019 | Adoption: At 4-week follow-up, 13% absolute increase (45% to 58%) in proportion of providers *Asking* about smoking to all patients in past month. Absolute increases in proportion of providers delivering treatment to smoking patient in the past month: 17% (55% to 72%), 19% (54% to 73%), 32% (29% to 61%), and 20% (25% to 45%) for *Advise, Assess, Assist, and Arrange,* respectively (all *ps* < .001) |
| Okoli 2018^§^  (USA) | Repeated cross-sectional | 3,669 psychiatric inpatients with primarily psychotic or depressive disorders | *Ask*, *Assist* with quit medication (NRT) and quit counseling | Services and mandatory assessments began January 1, 2016 | **Engage consumers**  Involve patients/consumers and family members  **Utilize financial strategies**  Alter incentive/allowance structures  **Change infrastructure**  Mandate change | Reach: At 12-month follow-up, 2% absolute increase in proportion of documented *Asking* all patients about tobacco use (93% to 95%, F (3, 12) = 7.39, *p* = .005). Significant positive linear trend over time for *Assisting* with counseling (F (1) = 18.59, *p* = .001), but no significant difference in average proportion of documented counseling rate (16% increase, 68% to 84%) for smoking patients. Significant positive linear trend in prescribing NRT over time (F (1) = 5.86, *p* = .032), but no significant difference in average prescribing NRT at longest follow-up (5% increase, 72% to 77%) for smoking patients. |
| Parker 2012^§^  (UK) | Repeated cross-sectional for Reach outcome  Cohort for effectiveness | 57 psychiatric inpatients and 53 psychiatric outpatients with depression, schizophrenia, bipolar, and “other” disorders | *Assist* with quit counseling, referrals, and quit medication (NRT) | 9 months | **Use evaluative and iterative strategies**  Conduct local needs assessment  **Train and educate stakeholders**  Conduct educational outreach visits  **Support clinicians**  Remind clinicians  Facilitate relay of clinical data to providers  Revise professional roles  **Engage consumers**  Intervene with patients/consumers to enhance uptake and adherence  **Utilize financial strategies**  Alter incentive/allowance structures  **Change infrastructure**  Change records systems | Reach: 8% absolute increase in recorded provision of NRT in medical record (3% to 11%) for inpatient smoker sample. No statistical test or p value reported. No baseline NRT data for community patients reported.  Effectiveness: Follow-up data collection period unclear. 7% of inpatients and 17% of community patients quit smoking; 7% of inpatients and 2% of community patients reduced smoking by at least 50%, and 25% of inpatients and 19% of community patients reduced by less than 50%. |
| Scharf 2011 (USA) | Repeated -cross- sectional | 30,908 psychiatric inpatients with psychotic, substance use, mood, and personality disorders | *Assist* with quit medication (NRT) | Smoking ban effective January 1, 2007 | **Train and educate stakeholders**  Distribute educational materials  Conduct educational outreach visits  **Support clinicians**  Remind clinicians  **Change infrastructure**  Mandate change  Change records system | Implementation: Per pharmacy records, Average monthly NRT units (unit = 1 patch, 1 piece of gum, 1 lozenge) significantly increased at 3-year follow-up (254 to 4,468 units, F(1, 11) = 67.76, p < .001), with gum or lozenge units increasing more than patch units (F(1, 22) = 26.62, p < .0001), and use of high-dose patches increasing more than low-dose patches over time F(1, 22) = 736.62, p < .0001), with more 21mg than 7mg patches prescribed (p < .0001). |
| Scheeres 2020 (USA) | Repeated cross-sectional | 8,983 psychiatric inpatients; diagnoses not reported. | *Assist* with quit medication (NRT or varenicline) | Smoking ban effective mid-December 2015 | **Use evaluative and iterative strategies**  Purposefully reexamine the implementation  Conduct local needs assessment  Obtain and use patients/consumers and family feedback  **Provide interactive assistance**  Provide local technician assistance  **Develop stakeholder interrelationships**  Develop academic partnerships  **Train and educate stakeholders**  Conduct educational meetings  **Change infrastructure**  Change physical structure and equipment | Reach: At 6-month follow-up, per pharmacy database, significant 5% increase in proportion of all patients during study period (11% to 16%, *p <* .001) receiving prescription quit medication (NRT patches, gum, or varenicline) (unadjusted OR = 1.58, 95% CI 1.42–1.76). |
| Schnoll 2023^§^ (USA) | Cluster RCT | 222 providers  610 psychiatric outpatients with psychotic, substance use, and other psychiatric disorders | *Advise* quitting, *Assist* with quit counseling, referrals, and quit medication (NRT, varenicline, bupropion) | 9 months | **Use evaluative and iterative strategies**  Assess for readiness and identify barriers  Audit and provide feedback  Conduct local needs assessment Obtain and use patients/consumers and family feedback  **Provide interactive assistance**  Provide local technician assistance  **Adapt and tailor to context**  Tailor strategies  **Develop stakeholder interrelationships**  Identify and prepare champions  Use an implementation advisor  **Train and educate stakeholders**  Conduct ongoing training  Provide ongoing consultation  **Support Clinicians**  Remind clinicians  **Change infrastructure**  Mandate change | Reach: At month 12 both sites increased *Assisting* smoking patient sample with any tobacco medication (NRT, bupropion, or varenicline), per medical record: ATTOC sites had 6% absolute increase (0% to 6%) and standard sites had 2% increase (6% to 8%)(no statistical test reported); NRT increased significantly (*p*=.001) by 4% at ATTOC sites (0% to 4%) and 1% at standard sites (1% to 2%); bupropion or varenicline increased significantly (*p* =.01) by 2% at ATTOC (0% to 2%) and 2% (5% to 7%) at standard sites. No significant site-time interaction for NRT only or bupropion/varenicline.  Adoption: Average frequency of staff-reported delivery of any 5A tobacco treatment increased significantly over time (β=4.77, 95% CI:3.83–5.70, *p*<0.001). No significant difference between groups over time. Absolute increases in proportion of staff delivering tobacco treatment not reported.  Effectiveness: Quit rates at month 12 were similar at ATTOC and standard sites, with 4% of patients in each reporting quitting. (β=0.43, 95% CI:0.21–0.83, *p*=0.01). |
| Wye 2017 (Australia) | Repeated cross-sectional | 2,898 psychiatric inpatients with schizophrenia, unipolar affective, bipolar, adjustment, borderline, other, substance use, neurodegenerative, and other disorders | *Ask, Advise* quitting, and *Assist* with quit medication (NRT) during hospitalization, *Assist* with quit medication (NRT) or referral to Quitline at discharge | 9 months | **Use evaluative and iterative strategies**  Audit and provide feedback  **Provide interactive assistance**  Provide local technician assistance  **Develop stakeholder interrelationships**  Identify and prepare champions  Conduct local consensus discussions  **Train and educate stakeholders**  Conduct ongoing training  Provide ongoing consultation  Distribute educational materials  Conduct educational meetings  **Support Clinicians**  Remind clinicians  **Engage consumers**  Involve patients/consumers and family members  **Change infrastructure**  Change records systems | Reach: At 5-month follow-up, 16% absolute increase in proportion of patient population during study period who had medical record documentation of *Asking* about smoking (36% to 52%, AOR = 2.39, 99% CI: 1.23 - 4.66), 8% increase in *Advice* (1% to 9%; AOR = 97.43, 99% CI: 31.03 - 306.30), 18% increase in *Assisting* with NRT (8% to 26%; AOR = 19.59, 99% CI: 8.17 -46.94), and 5% increase in *Assisting* at discharge (9% to 14%, AOR = 12.36, 99% CI: 6.08 - 25.14). |

*Note.* Due to being at critical risk of bias, Kanter-Bax et al., 2000 was included in tables and figures but not in the narrative synthesis. ^a^Population refers to those who were intervened upon and those on whom outcomes were collected. Many implementation strategies intervened on providers, but only collected patient-level outcome data (e.g, reach or effectiveness). The population on which outcomes were assessed (which was often patients) was the focus of risk of bias evaluations, in line with guidance from the Cochrane Group (34,35). ^b^Implementation strategies were categorized based on authors’ descriptions in the manuscript and authors’ responses to follow-up emails requesting additional information, when applicable. ^c^We only report data about cigarette smoking, although data on treatment for electronic nicotine delivery system use was also documented. Samaha et al., 2017 consulted for additional details about implementation strategy (36). ^d^Some details about implementation strategy derived from McFall et al., 2007 (37). ^§^Articles included in the limited quantitative synthesis of reach of *Ask* to all eligible patients and the reach of remaining *4A*s to smoking patients only.

ATTOC: Addressing Tobacco Through Organizational Change. CMS: Centers for Medicare & Medicaid Services RE-AIM: Reach, Effectiveness, Adoption, Implementation, and Maintenance
